# A fully integrated and automated 24-sample microfluidic system for sample-in-amplicon-out forensic analysis

**DOI:** 10.1038/s41378-026-01365-3

**Published:** 2026-07-08

**Authors:** Jun Nie, Yong Fan, Jichen Li, Jiaming Peng, Fuchun Li, Xiaoyue Luo, Lihuan Huang, Xiaochun Wan, Dehong Yan, Wenting Bu, Song Xu, Yongfan Men

**Affiliations:** 1 Guangdong Provincial Key Laboratory of Biomedical Optical Imaging Technology, Shenzhen Institutes of Advanced Technology, Chinese Academy of Sciences, Shenzhen, China; 2 Shenzhen Key Laboratory for Molecular Imaging, Shenzhen Institutes of Advanced Technology, Chinese Academy of Sciences, Shenzhen, China; 3 Nano Science and Technology Institute, University of Science and Technology of China, Hefei, China; 4 College of Life Sciences and Oceanography, Shenzhen University, Shenzhen, China; 5 Southern University of Science and Technology, Shenzhen, China; 6 Shenzhen Institutes of Advanced Technology, Chinese Academy of Sciences, Shenzhen, China; 7 Shenzhen Qixu Technology Co., Ltd, Shenzhen, China

**Keywords:** Nanofluidics, Electrical and electronic engineering

## Abstract

Rapid, reliable, and high-throughput DNA profiling is critical to modern forensic science. However, current analytical platforms are often limited by low sample throughput, insufficient system integration, and high operational costs. Here we report a fully integrated microfluidic system capable of performing automated, end-to-end forensic DNA analysis of 24 samples in parallel, with a coefficient of variation (CV) of merely 3.14%. The system incorporates sequential modules for cell lysis, magnetic bead-based DNA extraction and purification, multiplex PCR amplification, and product export for STR analysis within a sealed cartridge. Fluidic connectivity among four functional regions is precisely controlled via electromagnetic membrane valves, while automated reagent handling is achieved through solenoid and rotary valve systems. The platform yields high-quality STR amplicons from both buccal swabs and FTA cards, demonstrating complete concordance with conventional laboratory methods. Total 24-sample processing time is under 6 h, with a per-sample cost below $50. This technology offers a potential solution for high-throughput forensic DNA analysis.

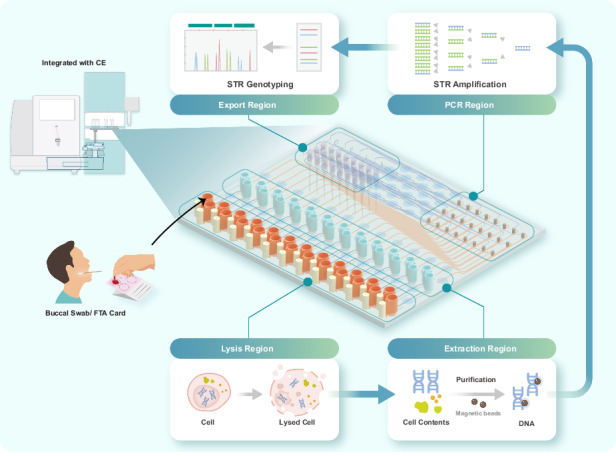

## Introduction

Forensic analysis refers to the scientific methods used to investigate and solve crimes, typically focusing on analyzing physical evidence to determine the truth^1^. The conventional overall process often encompasses: (1) sample cell lysis, (2) DNA (deoxyribonucleic acid) extraction and purification, (3) DNA amplification, (4) capillary electrophoresis, and (5) DNA profile generation^2^.

As the volume of biological evidence continues to increase, driven by rising demands in criminal justice, national DNA database expansion, and mass disaster victim identification, many forensic laboratories face significant challenges in processing large numbers of samples within a limited timeframe. Traditional workflows, often reliant on manual DNA extraction, amplification, and capillary electrophoresis-based STR (Short Tandem Repeat) genotyping^3–7^, are labor-intensive and time-consuming, resulting in persistent backlogs and delays in case resolution. Low-throughput systems also place significant burdens on personnel, increase costs, and prolong turnaround times, potentially delaying justice and impeding investigative outcomes^8^. Moreover, the complexity of forensic samples, which may be degraded, low in quantity, or involve DNA mixtures, further underscores the need for automated and high-throughput solutions. High-throughput and automatic platforms, by contrast, enable the simultaneous processing of tens to hundreds of samples, reducing per-sample costs, minimizing human error, and increasing laboratory productivity.

Each individual step in the forensic DNA analysis workflow, from cell lysis to STR genotyping, is supported by mature high-throughput instrumentation. For cell lysis, commercial systems such as the Precellys Evolution homogenizer (Bertin Technologies)^9^, TissueLyser II (Qiagen)^10^, and Bead Ruptor 96 (Omni International)^11^ allow for the simultaneous processing of 24 to 96 biological samples, including blood, swabs, and tissue. For nucleic acid extraction and purification, platforms like the KingFisher Flex (Thermo Fisher Scientific)^12^ and AutoMate Express (Applied Biosystems)^13^ offer automated, magnetic bead-based DNA isolation with capacities ranging from 13 to 96 samples per batch. High-throughput PCR amplification is widely enabled by thermal cyclers such as the VeritiPro and SimpliAmp (Applied Biosystems), as well as the QuantStudio 5 real-time PCR system, all of which support multiplex STR amplification in 96-well formats. For capillary electrophoresis and STR fragment analysis, advanced systems including the 3500xL Genetic Analyzer (Applied Biosystems)^14^ and SeqStudio Flex (Thermo Fisher Scientific) offer simultaneous processing of 8 or 24 capillaries, enabling parallel genotyping of STR loci at scale. The fragmented nature of these systems—each functioning independently and requiring manual transitions—significantly reduces operational efficiency and heightens the potential for cross-contamination.

Overcoming these limitations requires the development of fully integrated systems capable of performing sample-to-answer DNA analysis with minimal human intervention. Such systems not only increase processing capacity but also enhance data consistency, reproducibility, and legal admissibility. This is particularly essential in large-scale operations or scenarios involving degraded or mixed samples. In addition, the number of manual interventions, cross-contaminations, and the chance of mishandling could be significantly reduced.

However, existing research and commercially available integrated systems typically exhibit low throughput, often limited to only 1–4 samples per run. For example, Lounsbury et al. proposed a fully integrated polymeric one-channel microdevice that can realize rapid sample-in-PCR product-out with enzyme-based DNA preparation and PCR from swabs or blood samples^15^. Similarly, Roux et al. combine heat-activated enzymatic cell lysis and DNA release, infrared-mediated PCR amplification, and a 7 cm high-resolution STR amplicons separation into a four-channel plastic microfluidic to realize sample-in-answer-out analysis^16^. However, both prototype systems not only exhibit low throughput but also omit the nucleic acid purification step, which is critical for STR analysis as it enhances amplification efficiency, reduces background noise, and ensures accurate and complete genotyping^17,18^. Although systems like RapidHIT ID (Thermo Fisher) and ANDE 6C (ANDE Corporation) have improved portability and turnaround time, they are often constrained by limited throughput (1–5 samples per cartridge), high operational costs (several hundred dollars per sample), and a reliance on trained personnel^19–22^.

These limitations pose significant challenges to routine casework, large-scale database construction, and field-based forensic operations, particularly in resource-limited or time-sensitive scenarios. To address these gaps, an ideal solution must integrate all critical analytical steps, including cell lysis, nucleic acid extraction and purification, STR amplification, and genotyping, within a closed and contamination-free microfluidic platform. Such a system should operate with minimal human intervention, be accessible to non-specialist users, support scalable and parallel sample processing, and maintain a cost-effective profile suitable for widespread forensic development.

To overcome the persistent limitations of low throughput, insufficient system integration, and high cost in current forensic DNA workflows, we developed a fully automated, high-throughput microfluidic system capable of end-to-end genetic profiling. This 24-channel platform integrates cell lysis, magnetic bead-based DNA extraction and purification, microliter-scale multiplex PCR amplification, and the export of PCR products for CE-based STR analysis within a sealed microfluidic cartridge. The system is designed for parallel processing of 24 samples with minimal operator intervention and low contamination risk. It features four independently controllable functional units connected via electromagnetic membrane valves, with precise fluid handling enabled by solenoid and rotary valve control. Using buccal swabs and FTA (Fluid Transport Medium) cards as raw samples, the platform reliably extracted genomic DNA and produced complete and high-fidelity STR profiles with DNA template input ranging from 0.125 ng to 10 ng, consistent with conventional methods. With a total processing time of under 6 h and a per-sample cost below $50, this system offers a scalable and practical solution for both centralized forensic laboratories and field-based deployments.

## Results

### The microfluidic chip and system design

**Fig. 1:**
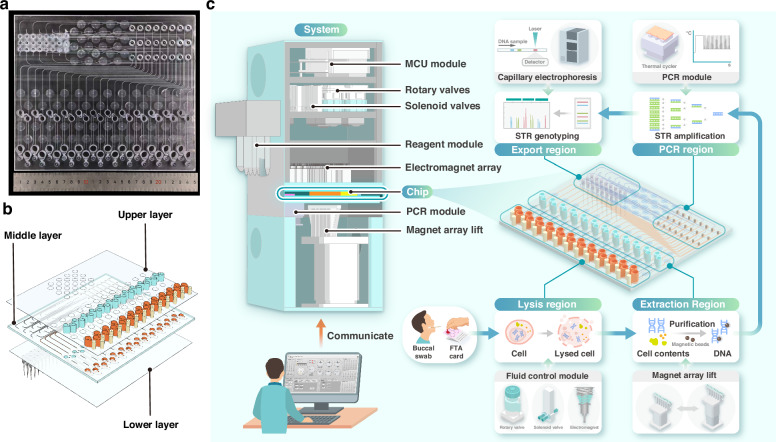
The microfluidic chip and system design. **a** Photograph of a 24-sample microfluidic chip designed for high-throughput forensic analysis. **b** Exploded 3D schematic showing the multilayer architecture of the chip. **c** Schematic illustration of the highly integrated and automated microfluidic system. This system integrates modules for programmable microcontroller unit, fluid control, magnetic beads control, temperature control, and user interface. Sample input from buccal swabs or FTA cards enters the lysis region, where cells are lysed and DNA are released. DNA is purified in the extraction region using magnetic beads. The purified DNA is then transferred to the PCR region for amplification, and the resulting products are analyzed in the export region via capillary electrophoresis and STR analysis. The system is centrally controlled via software and uses a multilayer chip design to support high-throughput, parallel processing of 24 samples

The chip used in the system is an integrated 24-sample microfluidic device designed for forensic analysis. The chip is rectangular in shape with a length of 250 mm, a width of 215 mm and a height of 5 mm (Figs. 1a and S1a). The microfluidic chip features a three-layer structure fabricated from polycarbonate (PC). It consists of a 0.5 mm top layer containing reaction chambers with upward protrusions and membrane valves, a 4 mm middle layer incorporating microchannels and valve cavities, and a 0.5 mm bottom layer with downward-protruding export outlets (Figs. 1b and S1b). This multi-layer configuration enables vertical chamber alignment, integrated fluidic routing, and compatibility with membrane valve actuation and high-resolution optical detection.

Figure 1c illustrates this fully integrated and automated 24-sample microfluidic platform for forensic DNA analysis, structured around a modular architecture consisting of both hardware and chip-level components. The schematic diagram in the left panel illustrates the overall system architecture, showing that the central instrument comprises multiple functional modules (Figs. 1c and S2a). The right panel of Fig. 1c shows that the chip is divided into four functional regions: the lysis region, extraction region, PCR region, and export region. Each region performs its specific function through dedicated functional modules integrated within the system. First of all, precise fluid routing, pressure control, chamber isolation, and interconnection across all four regions is achieved by the fluid control module consisting of six rotary valves (Figs. 1c and S2b), three solenoid valves (Figs. 1c and S2c), and 72 electromagnets (24 for three regions) (Figs. 1c and S2d). In the lysis chamber, cells from buccal swabs or FTA cards are lysed by chemical lysis buffer and heating to release the nucleic acids. In the extraction chamber, magnetic bead-based nucleic acid purification is carried out using a motorized magnetic array lift (Figs. 1c and S2e) that enables precise adsorption and dispersion of the beads. The amplification region is equipped with a rapid thermal cycling module (Figs. 1c and S2f) capable of fast temperature ramping for efficient STR amplification. Finally, in the export region, amplification products can be directly transferred to a standard capillary electrophoresis sequencing platform for downstream analysis. All of these functions are automatically controlled by a custom MCU (microprocessor control unit) -based module (Figs. 1c and S2g) located at the top of the system, which orchestrates operations through dedicated electrical circuits for each module. Additionally, a reagent module is equipped for storing and dispensing reagents required at each step (Figs. 1c and S2h). There is a chip insertion area that accommodates the microfluidic chip with integrated processing regions (Figs. 1c and S2i). Closed environment helps prevent contamination from external sources, which is critical in forensic cases where the integrity of the evidence must be preserved. Once the chip is inserted, the system operates in a fully automated manner, guided by a self-developed program based on LabVIEW that controls timing, temperature, fluid routing, and valve actuation (Figs. 1c and S2j). The entire process from raw sample input to STR genotyping output is completed with minimal human intervention, making the platform suitable for both centralized forensic laboratories and field-deployable scenarios.

### Chambers and channels

**Fig. 2:**
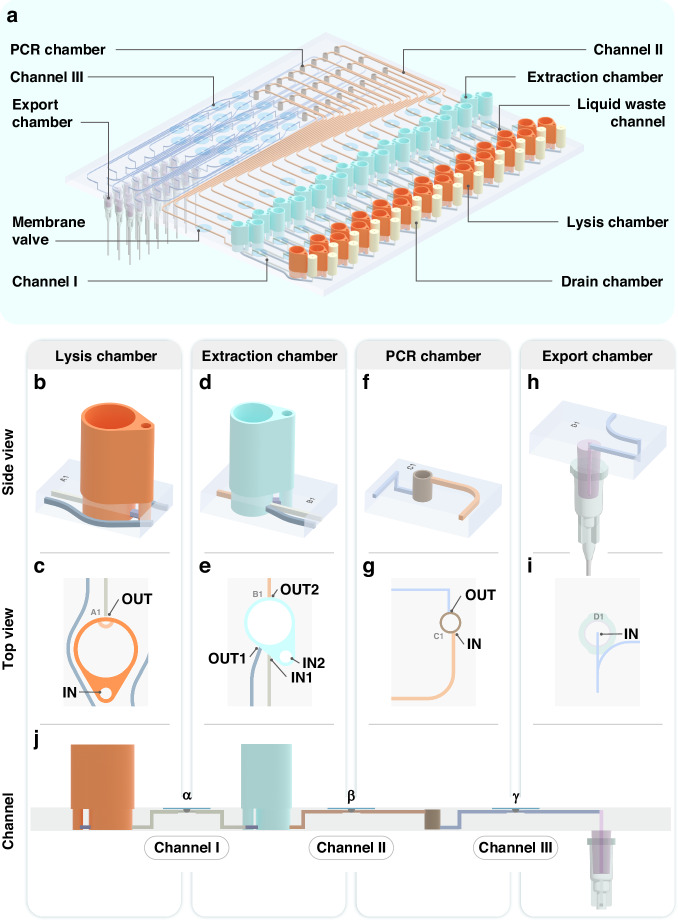
Structural design and functional layout of the 24-channel microfluidic chip. **a** Three-dimensional schematic of the chip, showing the sequential arrangement of the lysis, extraction, PCR, and export chambers. Interconnecting microchannels enable fluid transfer between regions: from the lysis chamber to the extraction chamber, then to the PCR chamber, and finally to the export chamber for downstream analysis. Integrated membrane valves and a liquid waste channel provide fluidic control and waste removal. Isometric and top views of the four main functional chambers: **b**, **c** lysis chamber, **d**, **e** extraction chamber, **f**, **g** PCR chamber, and **h**, **i** export chamber. **j** Cross-sectional view illustrating the vertical fluidic connectivity between the chambers via embedded microchannels

Figure 2 presents the elaborate spatial arrangement of functional chambers and micro-channels integrated on the 24-sample chip. Each sample pathway is independently assigned to a designated flow channel, enabling parallel processing across all 24 lanes. Microchannels interconnect four distinct functional regions mentioned in Fig. 1a, c in a sequential manner to facilitate automated fluid routing, with the lysis region located at the bottom of the chip, the extraction region in the center, the PCR region in the upper right corner, and the export region in the upper left corner. These four functional regions can be isolated by involving three sets of 24-channel membrane valves in between each two, which are controlled by mechanical electromagnetic solenoids. These three sets of electromagnetic solenoids valves are named as *α*, *β*, and *γ*. Therefore, different reactions can be performed independently in each region, and accurate amount of reaction products can be manipulated and transferred to next region for further reaction (Fig. 2a).

Each of these four major functional chambers has a distinct structural design tailored for its specific biochemical function. As shown in Figs. 2b, c, the lysis chamber is a vertical cylindrical structure with a single inlet and outlet, mounted above the microchannel network. From the top view, it adopts a teardrop-like layout with the liquid inlet (IN) positioned at the narrow end (diameters of 2 mm and heights of 18 mm, with a volume of 56.52 µL) for directional flow and sample positioned at the wide end (diameters of 9 mm and heights of 18 mm, with a volume of 1144.53 µL). This chamber is the initial site for cell disruption and nucleic acid release. Sample lysis reagents are introduced and mixed with biological samples, enabling effective cell membrane breakdown. After the chip is mounted onto the system, a vent port is integrated above the sample chamber’s sealing interface, allowing controlled exchange of air. This design enables precise regulation of air pressure within the lysis chamber, thereby facilitating the injection and transfer of liquids during sample processing. The chamber is connected to channel I via a membrane valve (interface α), allowing controlled transfer of lysate to extraction chamber for binding (OUT) (Figs. 2c, j). To prevent solid impurities being transferred along with the liquid, which could cause clogging or sample contamination, a dam structure was added at the outlet position.

As depicted in Fig. 2d, e, the extraction chamber is also cylindrical and vertical, similar in design to the lysis chamber but with a different size (diameters of 7 mm and heights of 18 mm, total volume 692.37 µL) to match the requirements of the biochemical reactions. This chamber is equipped with two inlets and two outlets for liquid, each serving distinct functions during the nucleic acid extraction process. One inlet (IN1) is connected to channel I, allowing upstream injection of lysate from the lysis chamber. The second inlet (IN2) is at the narrow end of the teardrop, specifically designed for the introduction of binding buffer, wash solutions, and elution buffer. To minimize the risk of cross-contamination between washing and elution steps, two separate outlets are incorporated. One outlet (OUT1) is connected to a dedicated drain chamber, allowing efficient removal of non-specific wash residues following the binding step. The other outlet (OUT2) is directly connected to channel II via a membrane valve (interface β), which links the extraction chamber to downstream modules (Figs. 2e, j). This outlet is exclusively used for transferring the eluted nucleic acids to the amplification chamber, ensuring the integrity of the purified sample for subsequent PCR analysis. This chamber performs nucleic acid purification, typically using magnetic beads. The design allows for bead capture via external magnets lift during binding, washing, and elution steps. Likewise, a vent port positioned above the extraction chamber is utilized post chip installation to enable effective pressure regulation and ensure smooth fluid handling during the extraction process.

Unlike the raised structures of the lysis and extraction chambers, the amplification chambers (Fig. 2f, g) are cylinders with cross-sectional diameter of 3 mm that embedded in the main body of the chip. This flat, embedded architecture facilitates efficient heat transfer during PCR thermal cycling. Once the chip is mounted onto the system, a heated lid capable of reaching 105 °C is pressed tightly against the upper surface of the amplification chamber to maintain a high temperature at the chamber top during the reaction. Simultaneously, a programmable thermal control module is in direct contact with the lower surface of the chamber, ensuring that the temperature of the PCR reagents closely matches the set temperatures throughout the amplification process. The PCR amplification chamber is designed with a top-positioned inlet to effectively prevent backflow during reagent loading. The outlet is located at the bottom of the chamber, facilitating the complete discharge of the amplified products and minimizing residual accumulation.

The export chamber, as shown in Fig. 2h, i, is an inverted funnel-like structure leading to an external needle-type outlet port, facilitating sample collection or downstream detection. It incorporates a vertical port with a bottom outlet and features a valve-controlled entry. This is the terminal chamber, used to collect and export amplified products to off-chip analysis systems, such as capillary electrophoresis, qPCR detection modules, or sequencing devices. It is connected via channel III (interface γ) to the PCR chamber (Fig. 2j). To enhance the compatibility of the chip, the PCR reaction region and product export region are arranged in a 3-row by 8-column format, perfectly matching the dimensions of a standard commercial 96-well plate or 8-strip tube. This design allows the PCR products to be directly transferred to the 96-well plate of a capillary electrophoresis STR analysis instrument for forensic analysis. A silicone membrane-sealed 96-well plate is first loaded onto the translation stage of a commercial capillary electrophoresis sequencer. The translation stage can be precisely aligned with the sample export zone of our system, where the outlet needle penetrates the silicone membrane and dispenses PCR products directly into the corresponding wells of the 96-well plate. Subsequently, the translation stage moves the loaded plate to the capillary sampling region of the sequencer, where the capillary needle pierces the silicone membrane and aspirates the sample for electrophoresis. Finally, STR profiles are automatically generated, achieving a fully integrated sample-in-profile-out workflow. The entire process is automated and enclosed without any manual intervention, and the system is compatible with the specifications of most commercial capillary electrophoresis sequencers (Fig. S3).

Based on the spatial distribution of the functional areas on the chip, the flow channels of the 24 samples will inevitably intersect to a great extent (Fig. S4a). To solve this problem, an innovative overpass flow channel structure was proposed (Fig. 2j). In short, where the flow channels must intersect, two flow channels are casted on the upper and lower surfaces of the chip, respectively, so that they do not affect each other. As shown in Fig. S4b, the flow resistance of channel I from the lysis to the extraction chamber (red curve) remained highly stable, with negligible deviation from the baseline across all channels, indicating excellent fabrication reproducibility in this segment. The flow resistance of channel III from the amplification chamber to the product outlet (green curve) also exhibited minimal variation, staying within ±5% of the normalized value, suggesting that this region of the chip maintained reliable dimensional control. In contrast, the flow resistance of channel II from the extraction chamber to the amplification chamber (blue curve) showed considerable variation. A gradual decline was observed from sample 1 to sample 24, with the normalized resistance decreasing to approximately 0.6 in the final sample. This non-uniformity may result from subtle differences in channel geometry in this section and could affect fluid delivery consistency during nucleic acid transfer. These findings underscore the importance of stringent control in microchannel fabrication, particularly in segments critical for fluid transferring. To ensure consistency among the 24 samples, it was necessary to set different parameters by the program, mainly the pressure and time parameters for liquid transfer, to ensure that channel II could transfer a quantitative amount of liquid. Experimental results showed that although the flow resistance of channel II varied significantly, it was still possible to achieve automated transfer of a certain amount of liquid by optimizing the system and setting appropriate parameters. 10 µL liquid can be automatically transferred in channel II among the 24 samples. The control of 10 μL ± 0.57 μL (mean ± SD, *n* = 3) liquid can be achieved by adjusting the parameters to reduce the flow resistance variation among 24-sample channels (Figs. S4c and S4d). The specific channel volumes and pressures are provided in Table S1. This indicates that the system has good adaptability to the flow resistance variations. By optimizing the pressure and time parameters through the program, the impact of flow resistance fluctuations on liquid transfer accuracy can be effectively compensated, thereby ensuring the consistency of operations among the 24 samples. This lays a solid foundation for the reliability and reproducibility of subsequent experiments.

### The valves and membranes

**Fig. 3:**
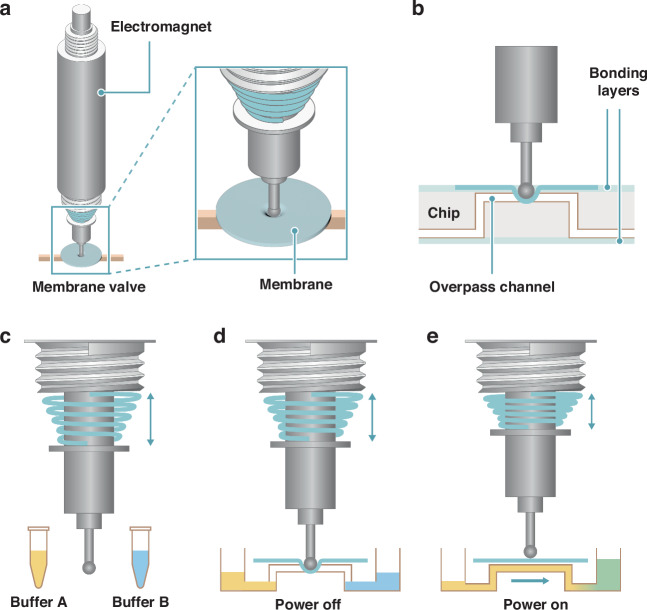
Illustration of the membrane valve. **a** Structure of the electromagnet-drived membrane valve. **b** Schematic of the valve acting on the overpass channel of the chip. **c**–**e** Different states of the valve for controlling the flow of buffer A and buffer B in the microfluidic channels

To control fluid communication between functional chambers, a membrane valve structure was integrated at the overpass region of the microfluidic channels (Figs. 2j and 3a, b). The membrane valve is controlled by a set of customized electromagnet devices. The electromagnetic membrane valve consists of a valve pit (an approximately hemispherical pit with a radius of 0.6 mm) and a silicone membrane (a circile with a diameter of 10 mm, thickness of 0.2–0.3 mm) attached on a 200 µm-deep cavity (a circile with a diameter of 12 mm), along with an externally designed spherical threaded valve head on the terminal of the electromagnetic core that controlled by the system (Fig. 3a, b).

There are three sets of 24-membrane valves in system, which are named as *α*, *β* and *γ* valves (Fig. 2j). The precise liquid injection and mixing of the 24 samples are automatically executed by the system via regulating the opening and closing of membrane valves. When the chip is not loaded into the system, the electromagnet spring remains in an unpressurized state, maintaining its default configuration. Upon loading the chip into the system and securing the clamping device, the electromagnet spring, in the de-energized state, undergoes a downward pressure of 2 N (Newtons), leading to spring compression. This compression subsequently induces deformation of the silicone membrane, with the 2 N force applied to the membrane being sufficient to occlude the flow channel, thereby preventing fluid communication between the two reaction chambers. When the electromagnet is energized, an upward force of 2.5 N is exerted on its spring, resulting in further compression. This action releases the pressure on the silicone membrane, causing the membrane valve to open and enabling fluid mixing. Such automated control of membrane valves ensures that each sample receives the precise volume of liquid at the designated position, with uniform mixing across all channels, thus further enhancing inter-sample consistency (Fig. 3c–e).

Different colors of solutions are used to demonstrated the features of valves. After injecting the yellow dye solution into the 24 lysis chambers with α valves closed, no visible leakage into adjacent extraction chambers or other compartments was detected during the 30-min observation period (Fig. S6a and Video 1). This confirmed the effective sealing capability of the *α* valves under static conditions. When the blue dye solution was injected into the extraction chambers with both *α* and *β* valves closed, no cross-chamber leakage was observed over 30 min (Fig. S6b and Video 1). This verified that the *β* valves could maintain isolation between the extraction chambers and other system components, keeping the adjacent lysis and extraction chambers in a fully segregated state. Upon opening the α valves and applying pressure to the lysis chambers, the yellow solution flowed through channel I into the extraction chambers. Real-time observation showed that the yellow and blue solutions gradually mixed, resulting in a uniform green solution within 2 min in all 24 channels (Fig. S6c and Video 1). This confirmed successful fluidic connectivity and efficient mixing between the adjacent chambers. Furthermore, we performed computational fluid dynamics (CFD) simulations to evaluate the liquid mixing process within the extraction chamber. The simulation results demonstrated that the cell lysate products and the binding solution for nucleic acid extraction could achieve sufficient and homogeneous mixing within 5 s even without air injection (Fig. S7).

Collectively, these results demonstrated that the membrane valves could precisely control liquid flow directions within the system, with consistent performance across all 24 channels. The absence of leakage in closed states and reliable fluidic communication in open states validated the robustness of the valve design and its suitability for parallel processing of multiple samples.

### The whole system operation and liquid manipulation

**Fig. 4:**
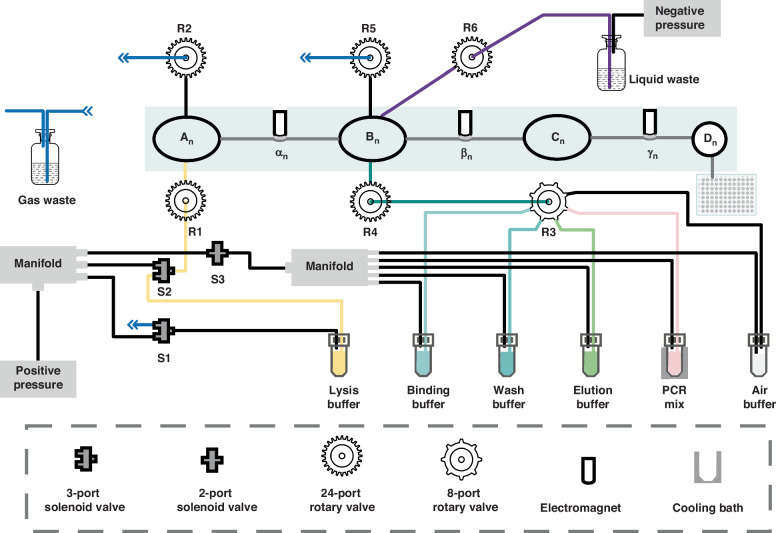
Schematic diagram of the system workflow. Reagents including lysis buffer, binding buffer, wash buffer, elution buffer, and PCR mix are delivered into appointed chambers through rotary valves (R1, R3, and R4) via a manifold system controlled by solenoid valves (S1–S3) and a multi-port rotary valve (R3). The process involves sequential steps through reaction chambers (A_n_ to D_n_), facilitated by positive and negative pressure sources. Rotary valves (R1–R6) guide fluid flow between chambers, while electromagnets and a cooling bath assist in bead-based nucleic acid capture and thermal control. Waste is directed to gas and liquid waste reservoirs

As illustrated in Fig. 4, all reagents and gases are manipulated by the fluid control module, mainly composed of six rotary valves and three solenoid valves. To enable parallel processing of 24 samples, we employed five 24-port rotary valves (24 inlets and 1 outlet) to control the fluidic connections to each chamber. To avoid cross-contamination between liquids, reagents used in the nucleic acid extraction steps were first switched by a 10-port rotary valve (10 inlets and 1 outlet), and then injected into different extraction chambers via a 24-port rotary valve. The lysis buffer (yellow color) is directed into the lysis chamber through a 24-port rotary valve (R1) via a 3-port solenoid valve (S1) for air pressure control and a 3-port solenoid valve (S2) for liquid control. In detail, clean air is introduced into the lysis chamber through R1 and S2 for bubbling after the lysis buffer is added. This bubbling facilitates thorough contact between the lysis buffer and the samples, enhancing cell disruption. Prior to lysis, another 24-port rotary valve (R2) is opened to release the pressure in the lysis chamber, ensuring sufficient buffer can be injected. Cells are lysed in lysis chamber (A_n_) under 65 °C for 20 min (Fig. S8a). After lysis, R2 is closed to seal the chamber, and air pressure applied via R1 and S2 pushes the lysate into the extraction chamber (Fig. S8b). All the other reagents are initially introduced into the chip via a 10-port rotary valve (R3) first. The binding buffer at port one (R3-P1), wash buffer at port two (R3-P2), and elution buffer at port three (R3-P3) are delivered to the extraction chamber via the third 24-port rotary valve (R4). Likewise, the PCR premix at port four (R3-P4) is introduced into the extraction chamber through R4, where it mixes with the eluted magnetic beads before being transferred to the amplification chamber. S3 and air buffer at port five (R3-R5) are applied for air pressure control. R5 is the vent for gas waste, and R6 is the outlet for liquid waste from extraction chamber after reactions (Fig. 4).

Before the DNA extraction step, the 24-port valve (R4) is opened to release pressure in the extraction chamber, enabling smooth reagent injection (Fig. S9a). Throughout the extraction and purification process, waste liquids are discharged via the 24-port valve (R5) (Fig. S9b). The elution product and PCR master mix are then driven into the amplification chamber by closing R4 and applying air pressure through R3 (Fig. S10a). After amplification, the PCR products are exported by introducing air pressure via R3 (Fig. S10b).

A customized gas source generated positive and negative pressure is first coarsely adjusted by two reducing vavles (IR1010-01B-A, IRV10-01BG, SMC) to match the working range of gas controllers, which provide precise adjustments of 0–1000 mbar (PRM-FOEM-1000, Fluigent) and -800-0 mbar (PRM-FOEM-N800, Fluigent). This integration offers numerous benefits in terms of efficiency, accuracy, and speed, all of which are critical when dealing with forensic samples.

### System function demonstration

#### System has high consistency

The experimental results demonstrated high consistency of nucleic acid extraction among the 24 channels of the microfluidic chip. To quantitatively evaluate the extraction consistency, the coefficient of variation (CV) of nucleic acid extraction efficiency across all 24 channels was calculated, yielding a low CV value of 3.14% (Table S2). This low CV value confirmed that there was no significant difference in extraction performance between different channels. These findings fully validate the excellent consistency and stability of the fluid control system integrated in the microfluidic device, which lays a solid foundation for the subsequent accurate and reliable formal PCR amplification and forensic DNA analysis (Figs. S11a and S11b).

The temperature consistency test results from Fig. S12 to Table S3 confirmed that the temperature control module maintained excellent uniformity across the entire 24-channel region. The SD values of actual temperatures at all key setpoints were ≤0.3 °C, indicating minimal temperature variation between different positions of the module. Meanwhile, during the steady-state holding period at each target temperature, the temperature fluctuation range was within ±0.1 °C, and no obvious temperature overshoot or fluctuation was observed during heating and cooling transitions. These results demonstrated the stable heating and cooling performance of the temperature control module, which provided a reliable temperature guarantee for the reproducibility of DNA amplification.

#### System has high compatibility

**Fig. 5:**
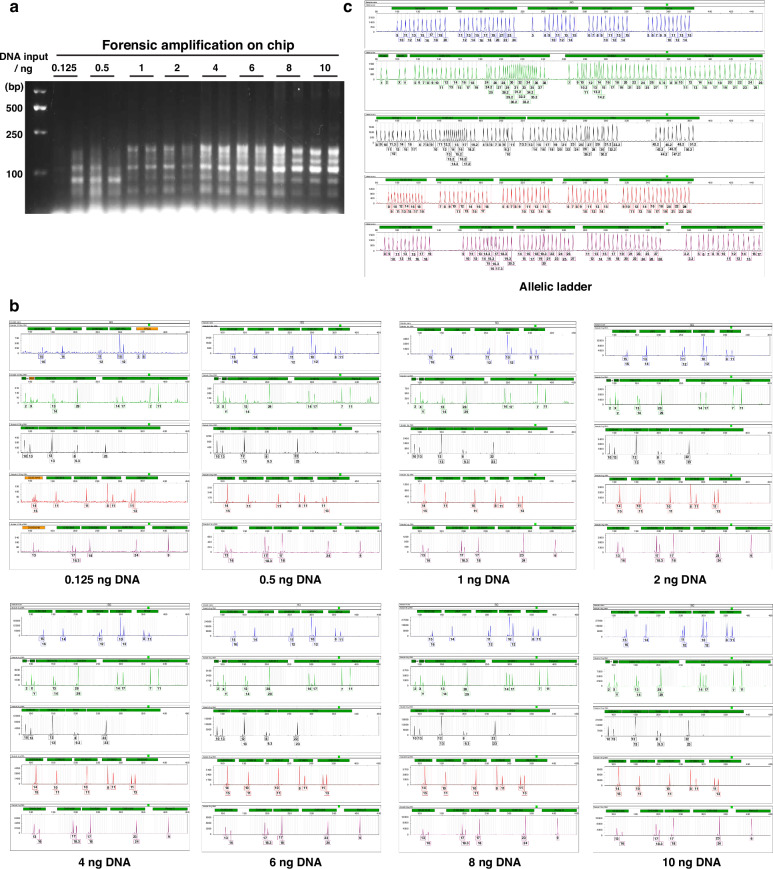
Performance validation of the integrated microfluidic system for forensic STR amplification and genotyping. **a** Agarose gel electrophoresis of on-chip amplified STR products from serial DNA dilutions (0.125–10 ng). The gel shows clear, concentration-dependent amplification products across all input levels, with no evidence of non-specific bands. **b** Capillary electrophoresis (CE) profile of the allelic ladder used for STR allele sizing and migration time calibration, confirming accurate fragment length assignment across all CODIS core loci. (c) CE STR genotyping profiles generated from serial DNA dilutions (0.125–10 ng) using the prototype system. Each column corresponds to a DNA input concentration, with rows representing CODIS core STR loci colored by fluorescent dye (FAM: blue; VIC: green; NED: black; TAZ: red; SID: purple). Allele numbers are annotated above each peak. All analyses were performed on an ABI 3500xL Genetic Analyzer with GeneMapper™ ID-X Software v1.5 (detection threshold: 500 RFU (relative fluorescence units); stutter filter: ≤15%)

The performance of the integrated microfluidic system for forensic STR analysis was systematically evaluated across a broad range of DNA input concentrations (0.125–10 ng), encompassing the variability of template quantities encountered in real-world forensic casework. Agarose gel electrophoresis of on-chip amplified products (Fig. 5a) demonstrated robust amplification efficiency across all input levels. Even at the lowest template concentration (0.125 ng), distinct, locus-specific bands were observed, indicating effective target enrichment without non-specific amplification or primer dimer formation. As DNA input increased, band intensity exhibited a dose-dependent increase, confirming consistent amplification kinetics and the system’s ability to handle both trace and high-concentration samples.

Capillary electrophoresis (CE) genotyping profiles (Fig. 5b) further validated the system’s reliability. Across all dilutions, all CODIS core STR loci produced detectable peaks (≥500 RFU) with accurate allele calling, no evidence of allele drop-out, and stutter peaks ≤10% of target heights, meeting the International Society for Forensic Genetics (ISFG) guidelines for reliable forensic typing. At the lowest input (0.125 ng), peak heights averaged 2150 RFU, and heterozygous peak balance exceeded 60%, demonstrating high sensitivity for low-template DNA. As DNA input increased to 10 ng, peak heights increased proportionally (to 9760 RFU) while maintaining profile integrity, with no peak saturation or spectral overlap (Table S4). Alignment with the allelic ladder (Fig. 5c) confirmed precise allele sizing and migration time calibration, ensuring compatibility with forensic DNA databases such as CODIS (Combined DNA Index System).

Across all DNA input concentrations (0.125–10 ng), the average peak-height balance (P) across loci ranged from 62% to 84% (Table S4). Even at the lowest input (0.125 ng), P remained above the 60% threshold recommended by ISFG guidelines, confirming uniform amplification efficiency. As DNA input increased, *P* values increased proportionally, reflecting improved amplification uniformity with higher template quantities. These results demonstrate that the prototype system maintains robust heterozygous peak balance across a broad dynamic range, supporting reliable allele calling for forensic applications.

These results collectively demonstrate that the prototype system exhibits robust forensic reagent amplification performance across a broad dynamic range of DNA inputs (0.125–10 ng). The system’s ability to maintain 100% amplification success, accurate genotyping, and consistent profile quality even with trace DNA addresses a critical challenge in forensic laboratories, where sample quantities vary widely from crime scene evidence to reference samples. Its compatibility with standard forensic workflows and databases further supports its potential for routine use, with implications for improving the efficiency and reliability of forensic DNA analysis.

### Optimization of the fully automated forensic STR amplification

A PCR premix without DNA template is introduced into the extraction chamber via a rotary valve, where it mixes with the magnetic beads retaining residual DNA. This mixture is transferred to the amplification chamber, where STR amplification is conducted directly in the presence of the beads. This streamlined approach eliminates the need for intermediate quantification or elution buffer splitting and is ideally suited for integrated microfluidic workflows.

According to the manufacturer’s instructions for the Huaxia Platinum PCR Amplification Kit, the optimal input DNA amount for STR amplification is 0.5–1.0 ng per reaction. DNA inputs below 0.25 ng may result in allelic dropout, whereas inputs exceeding 2 ng can produce overly saturated peaks and non-specific amplification. In our system, magnetic beads carrying the extracted nucleic acids are directly mixed with PCR premix and transferred into the amplification chamber. Therefore, it is critical to ensure that the total DNA input remains within the optimal range. To reduce template overload from buccal swabs or FTA cards and ensure optimal input concentration, a high-temperature elution step is first performed to remove excess nucleic acids through the waste outlet. To optimize the automated workflow, we then evaluated the residual DNA remaining on the magnetic beads following different numbers of elution steps. This analysis allowed us to determine the most appropriate elution protocol to ensure that the final DNA input for STR amplification falls within the optimal range. By fine-tuning the number of elution steps, we improved the efficiency and consistency of DNA recovery, laying a foundation for reliable downstream genetic profiling.

qPCR quantification showed that, for buccal swab samples, three consecutive elution steps using RNase-free water yielded residual DNA on the magnetic beads within the optimal concentration range for STR amplification (Fig. S13a–c). In contrast, for FTA card samples, two elution steps were sufficient to achieve a similar result (Figs. S13a, S13b and S13d). Based on these findings, we optimized the number of elution cycles in the automated DNA purification process accordingly.

To validate the system’s performance, buccal swab and FTA card samples were collected from two subjects. Automated lysis, extraction, and amplification were conducted using our integrated platform. STR profiling results demonstrated high-quality allelic calling with excellent reproducibility across replicates, confirming that the DNA extracted by the system is well-suited for downstream STR analysis (Fig. S14).

### 24 channels on chip are independent

To verify the channel independence and the absence of cross-contamination in the 24-channel microfluidic chip, a series of validation experiments was conducted. First, the isolation performance of adjacent channels was evaluated through interval liquid filling experiments. Specifically, dyed solutions were loaded into alternating even channels of the 24-channel microfluidic chip, including A2, A4,…, A24; B2, B4,…, B24; C2, C4,…, C24; and D2, D4,…, D24, while transparent water was added to the remaining odd channels. Visual observation of the chip after liquid filling showed no leakage of dyed solutions, and no mixing of dyed solutions into the adjacent clear water channels was detected, which directly confirmed the effective physical isolation between each channel (Fig. S15a).

In addition, PCR amplification experiments were performed to further validate the channel independence and eliminate the possibility of cross-contamination during the actual experimental workflow. Agarose gel electrophoresis was used to analyze the PCR amplicons obtained from all 24 channels. The electrophoresis results showed that clear and distinct bands with the expected size were only observed in the lanes corresponding to the channels loaded with samples. In contrast, no specific bands were detected in the adjacent blank control channels that were not loaded with samples. These results collectively demonstrated that there was no cross-contamination between adjacent channels throughout the entire process of sample processing and PCR amplification, confirming the complete independence of each channel in the 24-channel microfluidic chip (Fig. S15b).

## Discussion

High-throughput capacity, high integration, and full automation have become increasingly central to modern forensic DNA analysis, as they directly determine the speed, reliability, and scalability of case processing. With the growing demand for rapid investigative outcomes and large-scale forensic database construction, efficient sample processing has shifted from a desirable feature to a necessity. Systems that can automatically perform continuous workflows are particularly critical for reducing hands-on manipulation, improving reproducibility, minimizing contamination risks, and accelerating justice delivery. In this context, throughput and integration level have become key benchmarks for evaluating both conventional and emerging forensic technologies.

Despite continuous advances in forensic instrumentation, current DNA profiling strategies still face notable limitations. Many workflows remain fragmented, labor-intensive, and reliant on skilled personnel, leading to low throughput, high operational variability, and frequent sample backlogs. Although commercial rapid DNA systems and semi-automated workstations have improved processing speed, they often suffer from low parallelization capacity and insufficient integration across the entire analytical chain. High-throughput sequencing technologies, while powerful, require complex protocols, costly instruments, and intensive data analysis, restricting their routine use in most forensic laboratories. Collectively, these challenges highlight an urgent need for a fully integrated, automated, scalable, and cost-effective solution that bridges laboratory-level performance with field-accessible simplicity.

To address these gaps, we developed a fully integrated 24-channel microfluidic system that supports end-to-end forensic DNA analysis from raw biological samples to STR amplicon generation with minimal manual intervention. By consolidating DNA extraction and amplification into a single monolithic device, the platform effectively reduces manual handling, shortens turnaround time, lowers contamination risks, and improves overall workflow robustness. Compared with commercial systems such as RapidHIT ID and ANDE 6C, which cost several hundred US dollars per sample, our system achieves a per-sample consumable cost below $50 (excluding instrument depreciation, labor, and overhead), greatly reducing economic barriers for routine casework and large-scale database construction. In addition, unlike many research prototypes limited to 1–4 samples per run, our 24-channel integrated design adopts mature polycarbonate (PC) thermal compression bonding and precision CNC machining processes, which are compatible with mass production. The standardized modular layout and simplified fluidic control logic also reduce technical complexity in manufacturing and assembly, making the system more feasible for industrialization (Table S5).

Nevertheless, with the high-throughput and integration performance being presented, the current system remains at the proof-of-concept stage, and it has certain limitations that warrant further optimization. First, the system’s detection sensitivity has not been specifically enhanced, and its performance with highly degraded, low-template DNA or complex forensic samples with possible inhibitors, which are common in actual casework, remains to be improved. Second, the microfluidic chip fabrication process is still at the laboratory-scale machining rather than industrial large-scale mass production. Consequently, it is difficult to systematically evaluate and control run-to-run and chip-to-chip, or even batch-to-batch, variability, which may affect reproducibility in high-volume applications. Third, although the optimized system exhibited 100% success rate using standard specimens in controlled laboratory settings, real-world applications are often challenged by variable environmental conditions and complex sample types. Accordingly, the overall success rate under the combined influence of multiple external factors needs further investigation and validation.

Moving forward, improvements will focus on two core objectives: enhancing detection sensitivity and improving fabrication and performance uniformity. To boost sensitivity for low-concentration and degraded samples from real scenarios, we plan to replace the magnetic bead-based DNA purification with a direct amplification strategy, enabling immediate amplification of crude lysates without intermediate purification steps. In future work, we will perform extensive testing with real case samples, conduct repeated full-channel runs, and evaluate the system under variable environmental conditions to systematically assess its stability, failure rate, and amplification success rate for real-world forensic applications. We will also optimize microchannel geometries to reduce dead volume and shorten fluidic paths, further improving sample retention and amplification efficiency. To achieve better chip-to-chip consistency, we will refine fabrication processes to minimize flow resistance variation across the 24 channels, thereby enhancing fluid transfer uniformity without complex calibration. Future iterations will also explore on-chip STR separation and detection to achieve a truly sample-in–answer-out system, as well as integration of AI-driven process control and computer vision for intelligent monitoring and fault diagnosis. With continued engineering refinement, this high-throughput, cost-effective, and industrially adaptable microfluidic system holds great promise to become a transformative tool for next-generation forensic DNA analysis.

## Materials and methods

### Chip fabrication

The microfluidic chip was fabricated using a three-layer structure (upper, middle, and bottom) with PC (polycarbonate) as the base material, selected for its chemical inertness and optical transparency.

The 4 mm-thick middle layer, serving as the core fluidic network, was first subjected to precision micromachining using a computer numerical control (CNC) milling system (Accuracy ±5 μm). This process created microfluidic channels with a rectangular cross-section (0.6 × 0.6 mm) and different sizes of the base areas for different chambers at predefined locations. During the post-machining, the middle layer underwent deburring using a 0.5 mm-diameter nylon brush under controlled rotation (300 rpm) to remove burrs from channel edges, followed by ultrasonic cleaning in isopropyl alcohol (IPA) for 15 min at 40 kHz to eliminate residual debris.

The upper and bottom layers, each 500 μm thick, were aligned with the middle layer using a custom-built alignment jig featuring precision pin-and-hole guides (tolerance ±2 μm). Thermal compression bonding was performed in a vacuum hot press (vacuum degree <10 Pa) under optimized conditions: 125 °C temperature, 2 tons of pressure, and 5 h duration, ensuring hermetic sealing of interlayer interfaces without channel deformation.

Subsequent processing of the upper layer involved secondary CNC machining to form 200 μm-deep blind holes (diameter: 1.2 mm) for housing membrane valves at the crosspass channels. A secondary cleaning step was implemented, combining ultrasonic treatment in deionized water (15 min, 40 kHz) followed by nitrogen gas drying (0.3 MPa, 2 min) to prepare the surface for bonding.

Individually fabricated PC reagent chambers and universal Luer male adapter ports outlet pillars were bonded to the top and bottom surfaces of the chip, respectively, using a commercial glue. Finally, silicone membranes (thickness: 300 μm, Shore hardness: 50 A) were adhered to the blind holes on the upper layer, resulting in a sealed, planar chip surface with integrated fluidic control components (Fig. S1b).

### Software development

An upper computer software system was developed using LabVIEW to enable automated system control (Zenodo. 10.5281/zenodo.18503391). LabVIEW’s programming paradigm, which emulates instrument design logic through block diagrams, was employed to implement core functionalities, encompassing three key modules: nucleic acid extraction, nucleic acid amplification (PCR), and data storage/retrieval. The first two modules interface with lower-level hardware to support 24-channel parallel operations for nucleic acid extraction and amplification, while the third module facilitates experimental data management for laboratory personnel (Fig. S2j).

### Communication module

Real-time communication with lower-level devices is established via 485 serial protocols, with configurable baud rates and address assignments enabling multi-device control through a single serial port. The upper computer issues commands and dynamically adjusts operations based on real-time feedback from lower-level devices. A TCP interface is reserved to allow external clients to connect as a server, enabling remote invocation of control commands.

### Nucleic acid extraction module

This module orchestrates magnetic bead-based nucleic acid extraction processes, including sample lysis, bead binding, separation, washing, and elution, via integrated microfluidic chip control. Sequential reactions are executed through the synchronized control of gas pressure (regulated by a gas control board), rotational reagent and fluidic path selection, solenoid valve actuation, electromagnet-actuated fluid isolation, and heating plate temperature (managed via relays). Operators select up to 24 channels, configure process parameters, and initiate/pause operations. Real-time monitoring features include current process/channel indicators, adjustable pressure controls with real-time pressure displays, and lower-level device status readouts with pre-process command inputs.

### PCR module

Temperature, duration, and cycle number are controlled to execute PCR denaturation, annealing, and extension steps. After inputting reaction-compatible parameters, the module triggers PCR thermal cycling at the designated process stage. A real-time temperature curve is displayed, with curve data exportable as a document post-experiment for further analysis.

### Data storage and retrieval module

Experimental parameters are saved to date-named spreadsheet files via a “Save” function, with each entry timestamped by the minute. Previously saved data can be retrieved to streamline repeated experiments by reducing re-entry time.

### Channel and chamber design

The microfluidic channels of the chip were designed under the consideration of accommodating 24 parallel channels on a single chip while minimizing fluidic resistance. This design strategy ensures efficient integration of multiple reaction pathways without compromising the hydrodynamic performance of the system. The cross-sectional area of most of the flow channels on the chip is a 0.6 × 0.6 mm square, with a minimum interval spacing of 0.9 mm between two channels (Fig. S4a). However, in some areas, the spacing between channels has been adjusted to maximize the use of the chip surface area, as shown in the upper right corner in Fig. S4a.

The flow resistance inside the pipeline is calculated using the Darcy Weisbach equation^23^,

1 $$\Delta S=f\cdot \frac{L}{D}\cdot \frac{\rho {v}^{2}}{2}$$

where ΔS is the resistance loss along the flow path (Pa), *f* is the Darcy friction coefficient (dimensionless), *L* is the length of the flow channel (m), *D* is the diameter of the channel (m), *ρ* is the density of the liquid (kg/m³), *v* the velocity of the liquid (m/s). For laminar flow, the Darcy coefficient is determined by the following equation:

2 $$f=\frac{64}{{R}_{e}}$$

where the *Re* is Reynolds number. To assess the fluidic consistency of the microfabricated chip, the flow resistance of three key pathways, (1) from the lysis chamber to the extraction chamber, (2) from the extraction chamber to the amplification chamber, and (3) from the amplification chamber to the product outlet, was measured across 24 microfluidic channels. Flow resistance values were normalized against the value from Channel I to allow comparison across all paths (Fig. S4b).

The length of flow channels in different functional areas varies for different samples. In order to control the movement of a certain volume of liquid within the flow channel, we made estimation using the following formula (Fig. S16). For a two-dimensional pipeline with a circular cross-section area, according to the Poiseuille formula, the flow rate inside the pipeline can be expressed as^23^:

3 $$u\left(y,z\right)=\frac{16{{\rm{a}}}^{2}\Delta p}{{\pi }^{3}\mu L}\mathop{\sum }\limits_{n=1,3,5\ldots }^{\infty }\frac{1}{{n}^{3}}\left[1-\frac{\cosh \left(n\pi \frac{y}{2a}\right)}{\cosh \left(n\pi \frac{b}{2a}\right)}\right]\sin \left(n\pi \frac{z}{2a}\right)$$

where *a* is half length of pipeline, *b* is half width of pipeline, *μ* is the fluid viscosity, *L* is the length of the flow pipeline, Δ*p* is the pressure difference at both ends of the pipeline. The volume flow rate within microchannels can be calculated by the following equation:

4 $${Q}_{v}={\int }_{-a}^{a}{\int }_{-b}^{b}u\left(y,z\right){dydz}=\frac{b{a}^{3}\Delta p}{6\mu L}\left[1-\mathop{\sum }\limits_{n=1,3,5\ldots }^{\infty }\frac{1}{{n}^{5}}\frac{192}{{\pi }^{5}}\frac{a}{b}tanh\left(n\pi \frac{b}{2a}\right)\right]$$

which can be simplified as:

5 $${Q}_{v}=\frac{{a}^{3}b\Delta p}{6\mu L}\left(1-0.63\frac{b}{a}\right)$$

For a liquid with a volume of *V* in a pipeline, the flow time and flow rate are related as follows:

6 $$t=\frac{V}{Q}$$

We get appropriate parameters of ${\,\Delta P}_{1}$, ${t}_{1}$ for sample#1 by fluid experiments. As it is inconvenient

to control the volume of the liquid by $\Delta P$ using our LabVIEW software. So, we set *t*_1_ = *t*_i_, and from (5 to 6),

7 $$\frac{{\Delta P}_{1}}{{L}_{1}}\,=\,\frac{{\Delta P}_{i}}{{L}_{i}}$$

*t*_i_ is the control time of the fluid for sample #*i*, t1 is the control time of the fluid for sample #1. ${\Delta P}_{i}$ and ${L}_{i}$ represent the applied pressure and channel length of sample #*i*. It means the volume of the liquid for each sample can be controlled depending on their channel length.

The appropriate parameters of ${\,\Delta P}_{i}$ can be calculated. By repeating the methods above, we get ${\Delta \mathrm{P}}_{3}$, ${\Delta \mathrm{P}}_{4}$, ${\Delta \mathrm{P}}_{5}$…… And, for extraction and amplification steps. The designing and optimizing steps are same as depicted above.

The specific pressure and time parameters from empirical testing were provided in Table S1.

### The membrane valve design

In order to design valve pits, we conducted mechanical tests and fabricated valve pits with different radii on the test chip. The test chip is placed on a load cell. We used an injection pump to inject liquid uniformly into the chip, that the valve controls the on/off of the fluid. When the experiment begins, the infusion pump is turned on, and the fluid flows through the valve pit. At the same time, we move the electromagnet downwards towards the valve pit. As the electromagnet moves downwards, the force exerted by the electromagnet on the valve pit will increase. The valve pit is completely blocked, and the fluid flow is cut off. And, we record the magnitude of the pressure force as the sealing force. We tried different sizes of valve pits and conducted repeated experiments with silicone membranes of different thicknesses. The experimental results are shown in Fig. S5a. For the case of membrane with thickness of 0.3 mm, the sealing force ranges from 1.35 ± 0.13 N (mean ± SD, *n* = 3) to 3.37 ± 0.06 N (mean ± SD, *n* = 3). For the case of membrane with thickness of 0.5 mm, the sealing force ranges from 4.80 ± 0.13 N (mean ± SD, *n* = 3) to 10.30 ± 0.99 *N* (mean ± SD, *n* = 3). We chose the valve pit with radius of 0.6 mm because its appropriate size, meanwhile it requires less force to block the resized valve pit for both cases, which reduces the design difficulty of the electromagnet.

As the plugging force for sealing the valve pits varies with different elasticity of silicone membranes due to their mechanical properties. We also conducted mechanical tests using different types of elastic silicone membranes, using the method mentioned above. The test results are shown in Fig. S5b. For the case of the valve pit with radius of 1 mm, the sealing force ranges from 1.53 ± 0.37 N to 2.14 ± 0.42 N, (mean ± SD, *N* = 15). For the case of the valve pit with radius of 0.8 mm, the sealing force ranges from 1.60 ± 0.17 N to 1.90 ± 0.26 N, (mean ± SD, *N* = 15). For the case of the valve pit with radius of 0.6 mm, the sealing force ranges from 1.47 ± 0.20 N to 1.89 ± 0.18 N, (mean ± SD, *N* = 15). We chose the KRN-200 elastic silicone film model because it performs stably on different sizes of valve pits and requires relatively less blocking force.

### Membrane valve performance test

To verify the reliability of fluidic control and inter-sample consistency, water color experiments were performed using the 24-channel microfluidic system. The experiments aimed to evaluate the sealing performance of membrane valves and the effectiveness of fluidic connectivity between adjacent chambers.

#### Sealing test for *α* valves

All α valves (controlling fluid communication between lysis and extraction chambers) were set to the closed state via the system’s electromagnet control, with a 2 N downward pressure applied to the silicone membranes to ensure complete occlusion of channel I. A yellow dye solution (0.1% w/v tartrazine) was injected into each of the 24 lysis chambers at a controlled pressure of 200 mbar using the system’s gas-driven fluidic module. The chambers were observed for 30 min to check for leakage.

#### Sealing test for *β* valves

With the *α* valves remaining closed, all *β* valves (regulating fluid flow from extraction chambers to downstream modules) were closed. A blue dye solution (0.1% w/v brilliant blue) was injected into each extraction chamber at 150 mbar. The chambers were monitored for 30 min to detect any cross-chamber leakage.

#### Fluid connectivity and mixing test

The *α* valves were switched to the open state by energizing the corresponding electromagnets, which applied a 2.5 N upward force to release the silicone membranes, while *β* valves remained closed. A pressure of 55 mbar was applied to the lysis chambers for 10 s to drive the yellow solution through channel I into the extraction chambers. Real-time visual observation was conducted to record the mixing process of the yellow and blue solutions.

### Fluid control

#### Lysis buffer injection to the lysis chambers

The process of adding lysis buffer into the 24 lysis chambers was executed using a controlled fluidic system. Initially, solenoid valves S1 and S2 were set to the open state. An external positive pressure source (regulated to 200 mbar) was connected to the lysis buffer reservior via S1, enabling the lysis buffer to flow through S2 and rotary valve R1 into each of the 24 lysis chambers. After the primary buffer injection, S1 was closed, and filtered compressed air (100 mbar) was introduced into S2 for 15 s to purge any residual buffer in the connecting pipelines, ensuring complete transfer into the lysis chambers. Throughout this process, rotary valve R2 was maintained in an open state to allow continuous evacuation of air from the lysis chambers, preventing pressure buildup that could impede buffer entry (Fig. S6a).

#### Transfer of lysis products to extraction chambers

Following lysis, the transfer of lysis products from the lysis chambers to the extraction chambers was performed. Solenoid valves S1 and rotary valve R2 were closed to isolate the lysis chambers from external pressure sources and gas evacuation pathways. The α valve, which controls fluid communication between lysis and extraction chambers via channel I, was opened. A controlled positive pressure (55 mbar) was applied to the lysis chambers through S2, generating sufficient force to drive the lysis products through channel I into the corresponding extraction chambers. The duration of pressure application was optimized to 6 s to ensure complete transfer (Fig. S6b).

#### Extraction reagents injection into extraction chambers

The reagents in extraction procedure included binding buffer, wash buffer, and elution buffer, which were switched by a rotary valve (R3). Initially, *α* and *β* valves were closed to block fluid flow in both upstream and downstream directions of extraction chamber. Solenoid valve S3 was set to the open state, and rotary valve R3 was set to port 1 (R3-P1). An external positive pressure source (regulated to 150 mbar) was driven the binding buffer to flow through R3 and R4 into each of the 24 extraction chambers for 10S. After the primary buffer injection, S3 was closed, and R3 was switched to the air buffer port (R3-P5) allowed compressed air (75 mbar) to purge any residual buffer in the connecting pipelines for 10S, ensuring complete transfer into the extraction chambers. Throughout this process, rotary valve R5 was set to corresponding port of R4 to allow continuous evacuation of air from the extraction chambers, preventing pressure buildup that could impede buffer entry (Fig. S7a).

#### Extraction waste effluent discharge

After the extraction chamber incubated at 65 °C for 20 min, the reaction waste effluent needed to be discharge from the chip. First, the magnet array was raised to hold the magnetic beads to prevent washout. The *α* and *β* valve remained closed. Solenoid valve S3 was reopened, and rotary valve R3 was switched to port 5 (R3-P5). A controlled positive pressure (50 mbar) was applied to the extraction chambers through R4, generating sufficient force to drive the waste effluent to corresponding waste channels via R6. Meanwhile, an extra controlled negative pressure was applied to the output of waste bottle, the input of which was connected to a rotary valve R6. The duration of pressure application was optimized to 6 s to ensure complete transfer (Fig. S7b).

Repeat the above injection and discharge procedures using wash buffer and elution buffer, respectively.

#### PCR mix injection

As the same of binding buffer injection, the PCR mixing was injected by switching R3 to port 4 (R3-P4). Initially, *α* valve remained closed and *β* valve was opened. Solenoid valve S3 was set to the open state, and an external positive pressure source was driven the binding buffer to flow through R3 and R4 into each of the 24 amplification chambers. Then following with the PCR thermal cycling (Fig. S8a).

#### PCR product extraction

After finished the PCR reaction, *α* valve remained closed, *β* and *γ* valves were opened. Solenoid valve S3 was set to the open state, and R3 was switched to port 5 (R3-P5). An external positive pressure source was applied to push the production out (Fig. S8b).

### Computational fluid dynamics simulation

To verify the mixing effect between cell lysate and nucleic acid binding buffer, finite element fluid dynamics simulations were performed using ANSYS Fluent 2022 to characterize the liquid mixing process. A three-dimensional model of the nucleic acid extraction chamber was constructed, with geometric dimensions fully consistent with those of the actual microfluidic chip (see dimensions in Fig. 2d, e, Line 191). In the simulation setup, cell lysate was pre-loaded into the chamber, followed by injection of nucleic acid binding buffer at a pressure of 200 mbar to induce mixing. The viscosity coefficients of cell lysate and binding buffer were set to 1.5 cP and 1.1 cP, respectively. A species transport model was employed to describe the fluid mixing behavior. Simulations were conducted over a duration of 15 s, during which 250 μL of binding buffer was continuously injected into the extraction chamber pre-filled with 300 μL of cell lysate. Volume fraction contours of the lysate were subsequently visualized and analyzed using the simulation software.

### Biochemical reactions

Biochemical reactions on and off chip were conducted according to the protocols provided by the kit manufacturers.

#### DNA extraction on chip

The Mag-MK Buccal Swabs Genomic DNA Extraction Kit (Sangon Biotech, B518765) was used for cell lysis, DNA extraction, and purification in this system automatically. The test samples (buccal swab or FTA card) were first placed into the lysis chamber. SanMag magnetic beads (25 μL) were pre-embedded in the extraction chamber and adsorbed to the bottom of the extraction chambers via the magnet array lift platform. The chip was then placed into the system and sealed by the jig and fixture. The lysis buffer (150 μL PBS, 150 μL Buffer MACL, 75 μL Buffer MCL, and 10 μL Proteinase K, sequentially) was injected into the lysis chamber through the rotary valve R1, mixed thoroughly with the sample, and incubated at 65 °C for 20 min. Subsequently, 300 μL of the cell lysate was transferred into the extraction chamber. Then, 250 μL of the binding buffer (Buffer MA) was injected into the extraction chamber via rotary valves R3-P1 and R4. The magnet array lift was released to fully mix the mixture with the magnetic beads, ensuring sufficient binding of DNA in the sample to the magnetic beads. The magnetic beads were adsorbed again, and the supernatant was discharged out of the chip through the rotary valve R6. Next, 600 μL of wash buffer (70% ethanol) was injected into the extraction chamber via rotary valves R3-P2 and R4. The magnetic beads were released, and after thorough mixing, the magnetic beads were adsorbed in the same manner, and the supernatant was discharged out of the chip through R6. The above washing cycle was repeated. The heating module of the extraction chamber was turned on to 55 °C, and air was blown into the extraction chamber via rotary valves R3-P5 and R4 to dry the ethanol on the surface of the magnetic beads. The magnetic beads were adsorbed, and 50 μL of elution buffer (RNase-free water) was added into the extraction chamber through rotary valves R3-P3 and R4. The magnetic beads were released again, incubated at 65 °C for 5 min, and intermittently aerated for mixing. The magnetic beads were adsorbed, and the number of elution cycles was determined according to the total amount of residual DNA on the magnetic beads. Finally, 20 μL of PCR premix was added into the extraction chamber via rotary valves R3-P4 and R4 and then injected into the amplification chamber together with the magnetic beads for nucleic acid amplification.

#### Consistency test of DNA extraction in 24 channels of the chip

Prior to formal polymerase chain reaction (PCR) amplification, the consistency of nucleic acid extraction across the 24 channels of the microfluidic chip was evaluated to verify the reliability of the integrated system. All experiments were performed with 24 samples undergoing automated nucleic acid extraction simultaneously in parallel channels, following the standard operating procedure (SOP) of the integrated microfluidic system. Each sample channel was tested at least three times to ensure the reproducibility of the experimental results, and all tests were conducted under the same experimental conditions (including reaction temperature, incubation time, and fluid flow rate) to eliminate potential confounding factors.

#### Stability Test of the temperature control module and DNA amplification

To evaluate the stability of the temperature control module in the integrated microfluidic system, the PCR amplification program was set as follows: initial denaturation at 95 °C for 60 s; 30 cycles of denaturation at 95 °C for 3 s, annealing at 59 °C for 16 s, and extension at 65 °C for 29 s; final extension at 60 °C for 5 min, followed by holding at 4 °C.

To complementarily verify the temperature uniformity and heating and cooling stability of the heating module, an electric heating cavity was employed for temperature measurement experiments. The electric heating cavity was closely attached to the surface of the temperature control module to ensure accurate temperature detection. Temperature measurements were performed at the key set temperatures of the PCR program (95 °C for denaturation, 59 °C for annealing, 65 °C for extension, and 60 °C for final extension), as well as during the heating and cooling transitions between these temperatures.

Three independent measurement replicates were conducted at each set temperature, and the temperature values at 5 different positions (covering the central and edge regions corresponding to the 24 channels) of the temperature control module were recorded simultaneously. The temperature uniformity was evaluated by calculating the mean value and standard deviation of the measured temperatures at each setpoint, while the heating/cooling stability was assessed by monitoring the temperature fluctuation range during the steady-state holding period at each target temperature and the temperature transition rate between adjacent set temperatures.

#### Agarose gel electrophoresis

For agarose gel electrophoresis, a 2% agarose gel was prepared with Tris-acetate-EDTA (TAE) buffer, and 5 μL of each amplification product (mixed with 1 μL of 6× loading buffer) was loaded into the gel wells. Electrophoresis was performed at 120 V for 30 min, followed by visualization under a UV transilluminator (Bio-Rad GelDoc XR+). A 100 bp DNA ladder was used as the molecular weight marker to confirm the size of the amplification products.

#### CE sequencing

A genetic analyzer (e.g., ABI 3500xL) was employed with optimized parameters for forensic DNA analysis: capillary length of 36 cm, separation voltage of 15 kV, running temperature of 60 °C, and detection wavelength of 490 nm. Each concentration gradient sample was tested in triplicate to ensure experimental reproducibility, and all experiments were conducted under the same environmental conditions to avoid external interference on the temperature control module.

In addition, the capillary electrophoresis profiles and agarose gel electrophoresis images of samples with concentrations ranging from 0.125 ng to 10 ng were systematically analyzed. CE profiles were used to evaluate the correlation between input nucleic acid concentration and amplification product performance (signal intensity, peak shape) as well as amplification specificity, while agarose gel images were employed to confirm the integrity and size uniformity of the amplification products, excluding non-specific amplification and product degradation.

#### Elution time determination for beads PCR

To quantify the total residual DNA on magnetic beads after different elution cycles, quantitative polymerase chain reaction (qPCR) was employed as previously mentioned^24^. A standard curve correlating cycle threshold (*C*_t_) values with template quantity was first constructed using cDNA of known concentrations. Briefly, serial dilutions of the human genomic cDNA (Quantagene Inc.) extracted from HeLa cells (ranging from 0.125 to 13 ng) were prepared, and qPCR amplification was performed for each dilution in triplicate using house-keeping GAPDH gene as the template. The reaction system (20 μL total volume) contained 10 μL of TaqMan® Fast Advanced Master Mix (2X, Thermo Fisher Scientific Inc.), 1 μL of TaqMan® Gene Expression Assay (FAM dye) mix (20X) (GAPDH gene, Hs02758991_g1, Thermo Fisher Scientific Inc.), 1 μL of template solution, and 8 μL of RNase-free water (Takara Inc.). Amplification was carried out on a commercial qPCR instrument (Q225, Quantagene Inc.) with the following thermal profile: an initial UNG incubation (2 min at 50 °C) and polymerase activation (20 s at 95 °C), followed by 40 two-step cycles (1 s at 95 °C and 20 s at 60 °C). The standard curve was generated by plotting the mean *C*_t_ values of the triplicate reactions against the logarithm of the known template concentrations, with the linear regression equation derived to quantify unknown samples (Figs. S13a and S13b and Table S6).

8 $${C}_{t}=a{\log }_{10}(input)+b$$

Where *C*_t_ is the cycle threshold, input is the amount of the standard DNA samples. Therefore, the unknown samples can be calculated as:

9 $$input={10}^{\frac{{C}_{t}-b}{a}}$$

For each elution cycle, magnetic bead samples were collected and directly mixed with PCR Master mix without template mentioned above, and then subjected to qPCR under the same reaction conditions as the standard curve. The total DNA quantity in each sample was calculated by substituting the measured *C*_t_ values into the standard curve equation, enabling comparison of residual DNA levels across different elution cycles (Figs. S13c and 13d and Tables S7 and S8).

#### Forensic analysis

The Huaxia™ Platinum PCR Amplification Kit (Thermo Fisher Scientific, Cat# A31323) was used for nucleic acid amplification. The reaction system (20 μL total volume) contained 8 μL of Master Mix, 8 μL of Primer set, and 4 μL of elution buffer with magnetic beads. Amplification was carried out with the following thermal profile: an initial incubation (1 min at 95 °C), followed by 30 three-step cycles (denaturation for 3 s at 94 °C, annealing for 16 s at 59 °C, and extension for 29 s at 65 °C) and a final extension (6 s at 60 °C). The amplification products were exported through the outlet chamber and could be directly loaded onto a commercial capillary electrophoresis sequencer (ABI 3500). All the above operations were automatically completed within the system via pre-set programs.

To evaluate the accuracy and stability of the system, buccal swab and FTA card samples were collected from two subjects. For buccal swab collection, sterile nylon-flocked swabs (Munkcare) were used to rub the inner cheek of each subject for 30 s, followed by immediate storage in sterile tubes containing 1 mL of preservation buffer. FTA card samples were prepared by spotting 50 μL of venous blood onto Whatman FTA cards, which were air-dried at room temperature for 2 h and then stored in sealed bags with desiccants until use. All samples were processed following the aforementioned experimental procedures and parameters for nucleic acid extraction, amplification, and capillary electrophoresis sequencing.

The accuracy of the system was assessed by comparing the sequencing results with the reference sequences of the target nucleic acid regions. The stability was evaluated based on the consistency of sequencing results across multiple replicate experiments (*n* = 3) for each sample type from both subjects.

Data analysis was performed using GeneMapper™ ID-X Software v1.5 (Thermo Fisher Scientific, Cat# A27884). Prior to experimentation, decontamination of microfluidic channels was achieved using DNA AWAY surface decontaminant (Thermo Fisher Scientific, Cat# 7010) (room temperature, overnight), 75% ethanol and sterile water, followed by surface modification with bovine serum albumin (Sigma, B2064) (37 °C, 1 h) to minimize nonspecific adsorption.

#### Channel independence test

Automated interval liquid filling was conducted using the integrated system. Colored reagents were alternately loaded into 12 channels, while the remaining 12 interleaved channels were filled with transparent water. The chip was then removed from the system and incubated at room temperature for over 1 h. Visual inspection was performed to confirm the absence of liquid mixing or leakage between adjacent channels.

To further validate channel independence under operational conditions, forensic PCR amplification was performed on the interleaved channels. High-concentration DNA samples were loaded into alternating channels, while adjacent channels were used as blank controls. Following on-chip nucleic acid extraction and amplification, the products were analyzed by agarose gel electrophoresis.

#### TFRC amplification

To verify the uniformity of nucleic acid extraction, the extraction products from the 24-channel chip were collected, and nucleic acid amplification was performed using the human reference gene TFRC (Transferrin receptor) as the template.

## Supplementary information

Membrane Valve Performance

ethics approval
