# Supplementary material for: A fully integrated and automated 24-sample microfluidic system for sample-in-amplicon-out forensic analysis
Source: Microsyst Nanoeng. 2026 Jul 8;12:258. doi: 10.1038/s41378-026-01365-3 (PMC13341781; doi:10.1038/s41378-026-01365-3)
Supplement: Supplementary file 2 — ethics approval [file 41378_2026_1365_MOESM2_ESM.docx]

Figure S1 Flowchart of the Chip fabrication, which includes seven core steps. Step 1, machining the main channels into the chip main body (Top view of the chip design, with all the chambers labeled); Step 2, aligning the top and bottom thin layer onto the chip main body, forming a three-layer sandwich structure; Step 3, perfoming the thermal compression-bonding procedure, forming the enclosed channels; Step 4, with the thin layer bonded, arrays of blind holes for the chambers and membranes were drilled; Step 5 several cylindrical chambers and ports were fabricated and bonded onto the blind holes; Step 6, arrays of silicone membrane were bonded onto the blind holes; Step 7, the whole chip was completed.


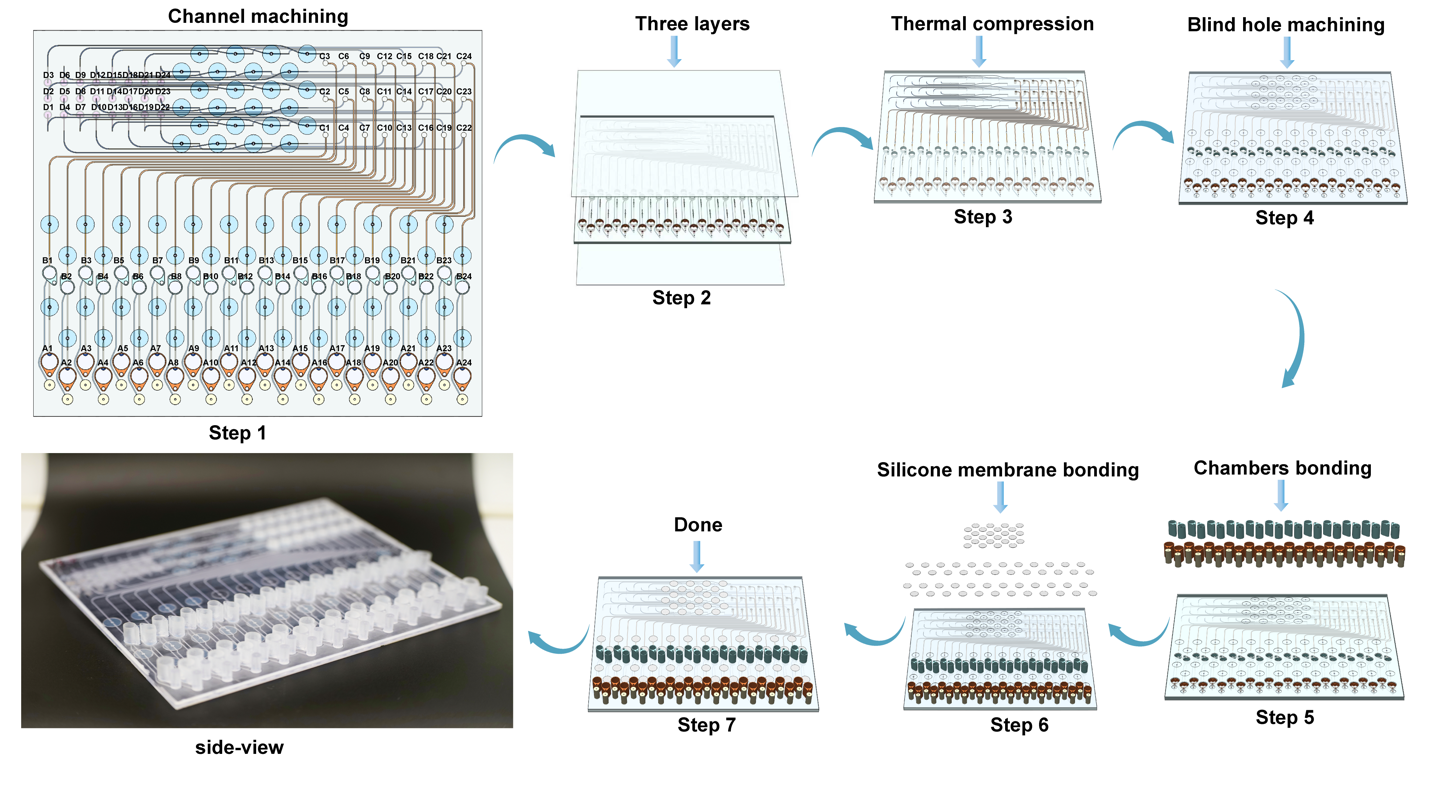

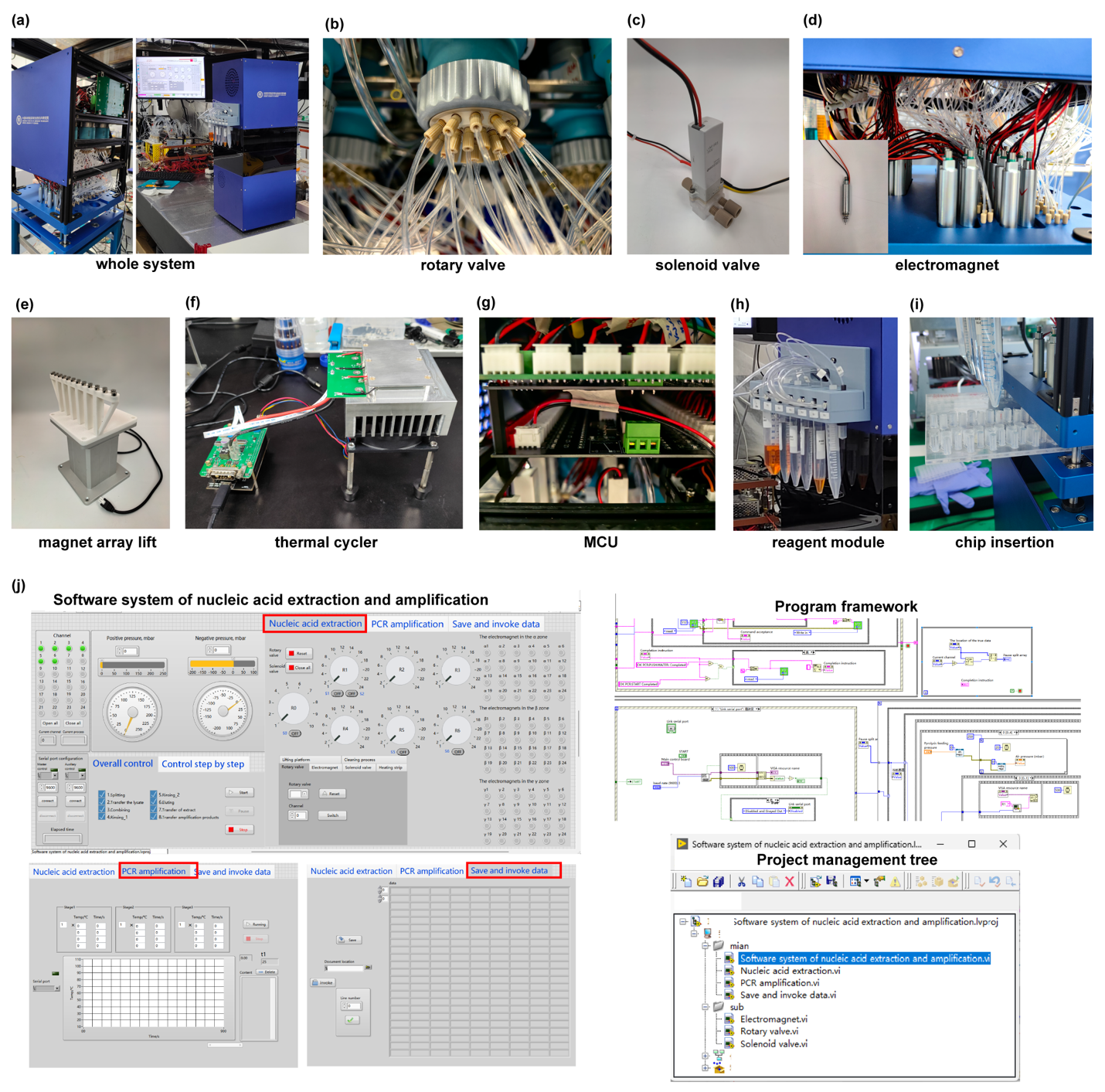


Figure S2 Physical pictures of the entire system and each module (a) Whole system. left panel: assembling state. Right panel: operating state. (b) rotary valve fully connected with tubings (c) soleniod valve (d) electromagnet array (e) magnet array lift. (f) thermal cycler (g) MCU. (h) reagent module (i) chip insertion area (j) LabVIEW program, including the interface and the framework.

Figure S3 This prototype enables seamless integration with commercial capillary electrophoresis instruments, achieving the ultimate **sample-in-profile-out** workflow. (a) **Sample in**: The 24-sample microfluidic chip is loaded with buccal swab samples, while a silicone membrane-sealed 96-well plate is positioned on the motorized translation stage of the capillary electrophoresis (CE) instrument. (b) **Amplicon extraction**: After on-chip nucleic acid extraction and amplification, the motorized translation stage aligns the 24-sample chip with the 96-well plate. The stage lifts to enable extraction of the amplicons from the chip’s extraction chambers directly into the corresponding wells of the 96-well plate. (c) **Amplicon in CE**: The motorized translation stage transports the 96-well plate into the CE instrument, where the amplicons are transferred to the capillary array for electrophoretic separation. (d) **Profile out**: The capillary array performs amplicon sampling, and the CE instrument generates the final STR profile, completing the fully automated, closed-tube sample-in-profile-out workflow.


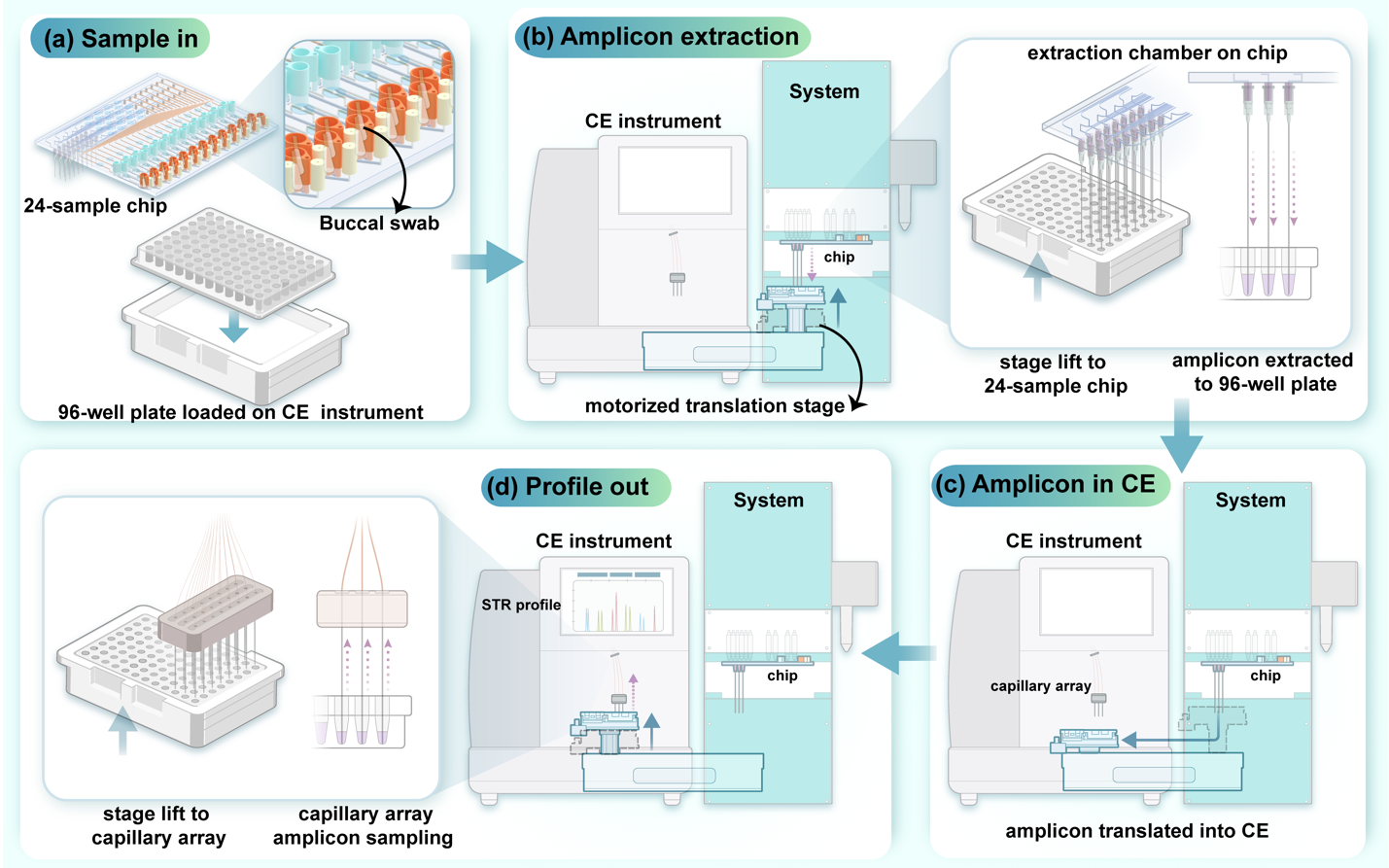


Figure S4 The flow channels on the chip (a) The cross-sectional area and the minimum interval distance of the channels on the chip. (b) The normalized flow resistance of the channels on the chip. (c) 10 µL liquid can be automatically transferred in channel II among the 24 samples. (d) The control of 10 μL ± 0.57 μL (mean ± SD, n=3) liquid can be achieved by adjusting the parameters to reduce the flow resistance variation among 24-sample channels. Error bars represent the standard deviation of triplicate measurements for each channel.


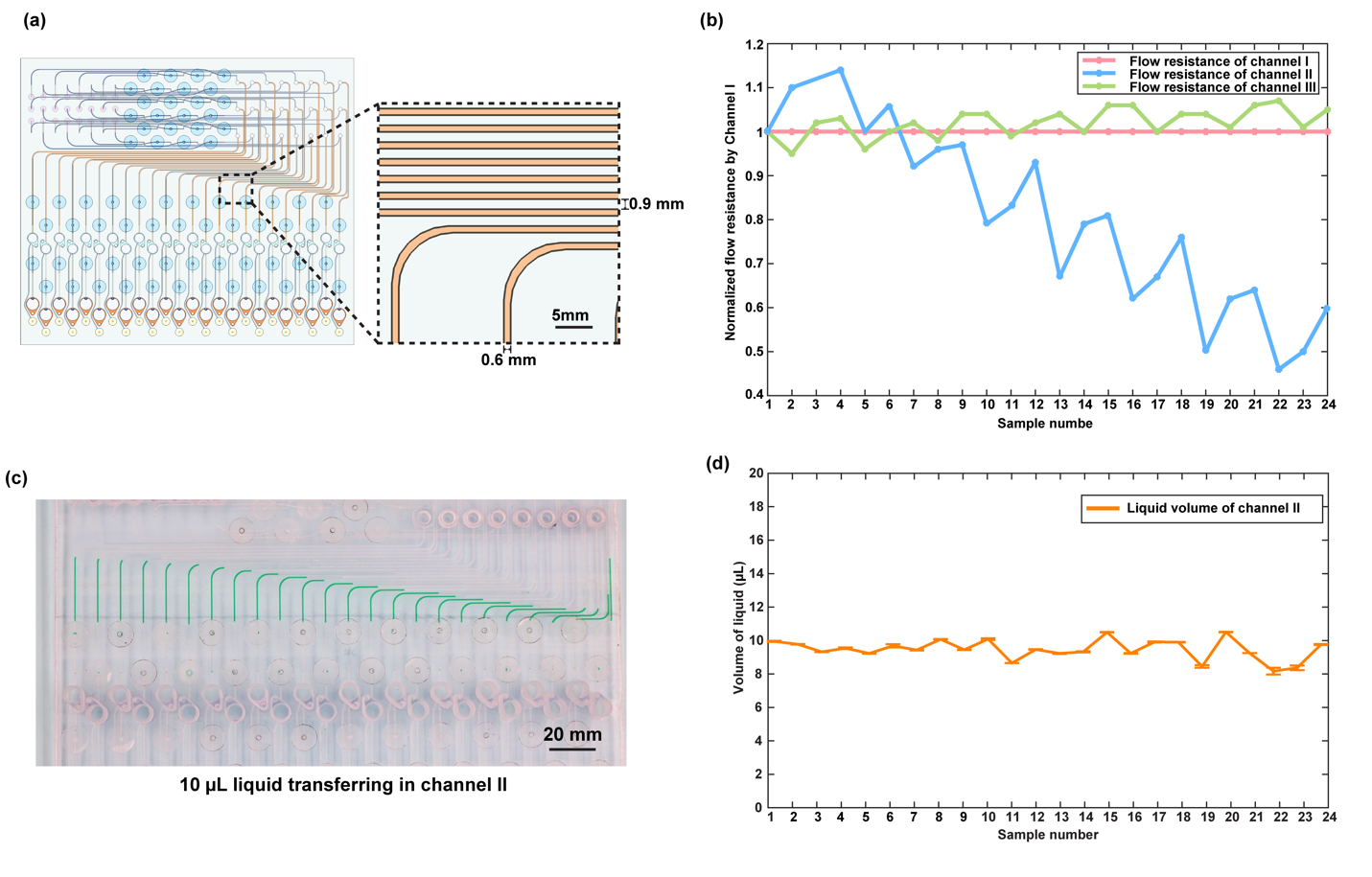

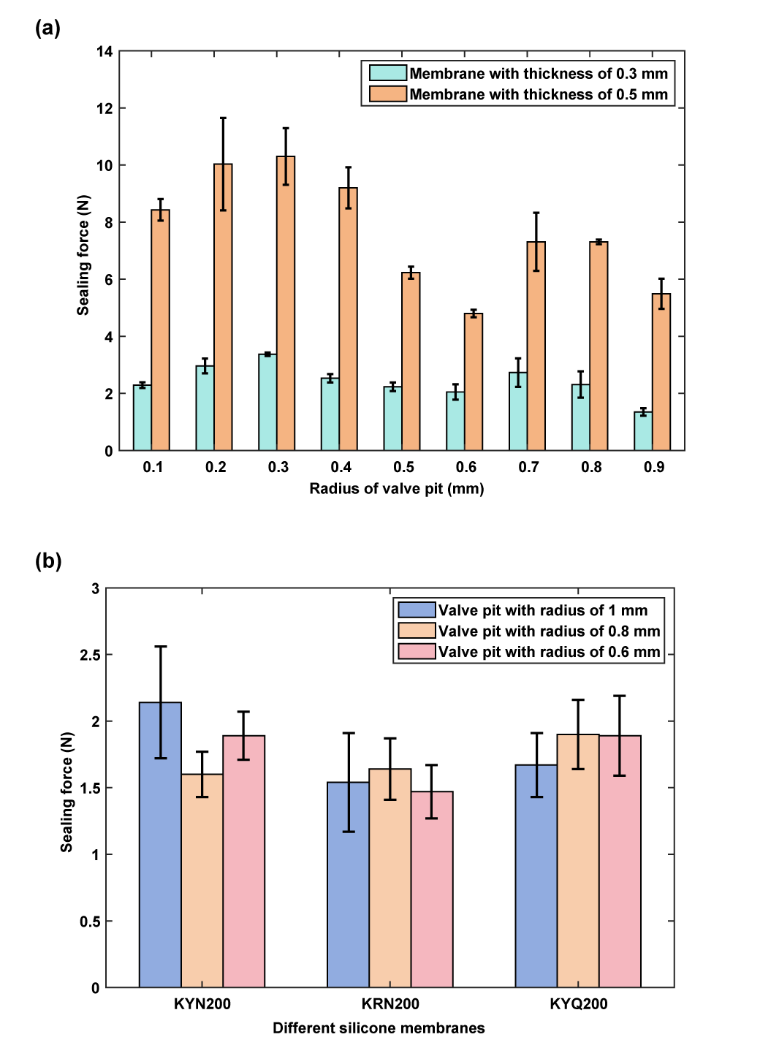


Figure S5 (a) Electromagnetic rebound force required for sealing valves with different radii and membrane thicknesses (b) The force required for sealing valve pits with different radii of silicone film with different types

Figure S6 Precise liquid injection and mixing of the 24 samples are automatically accomplished by the system through controlling the opening and closing of the membrane valves. (a) Yellow liquid was injected in lysis chambers while closing α valves (b) Blue liquid was injected in extraction chambers while closing both α and β valves. (c) Yellow liquid was transferred into extraction chambers, mixed with blue liquid, and turned into green while opening α valves and closing β valves.


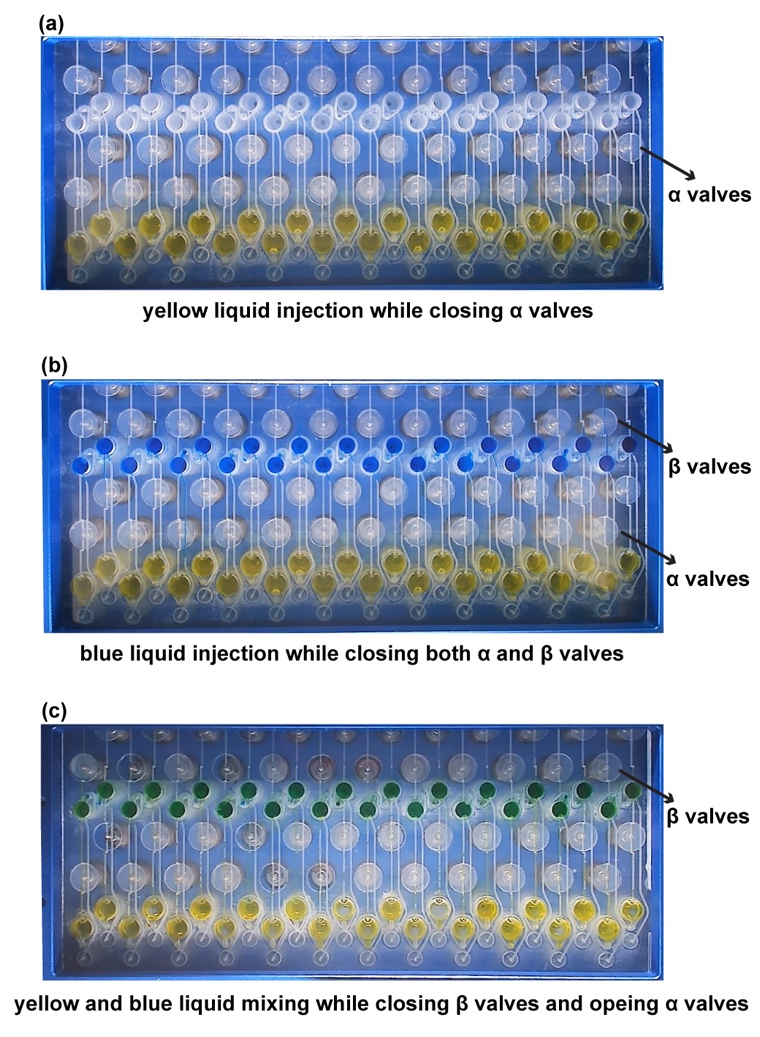


Figure S7 Computational fluid dynamics simulations for evalution of the liquid mixing process within the extraction chamber within 11 seconds. (a-c) Variation in the volume fraction of lysate in the extraction chamber with injection time of the binding buffer.


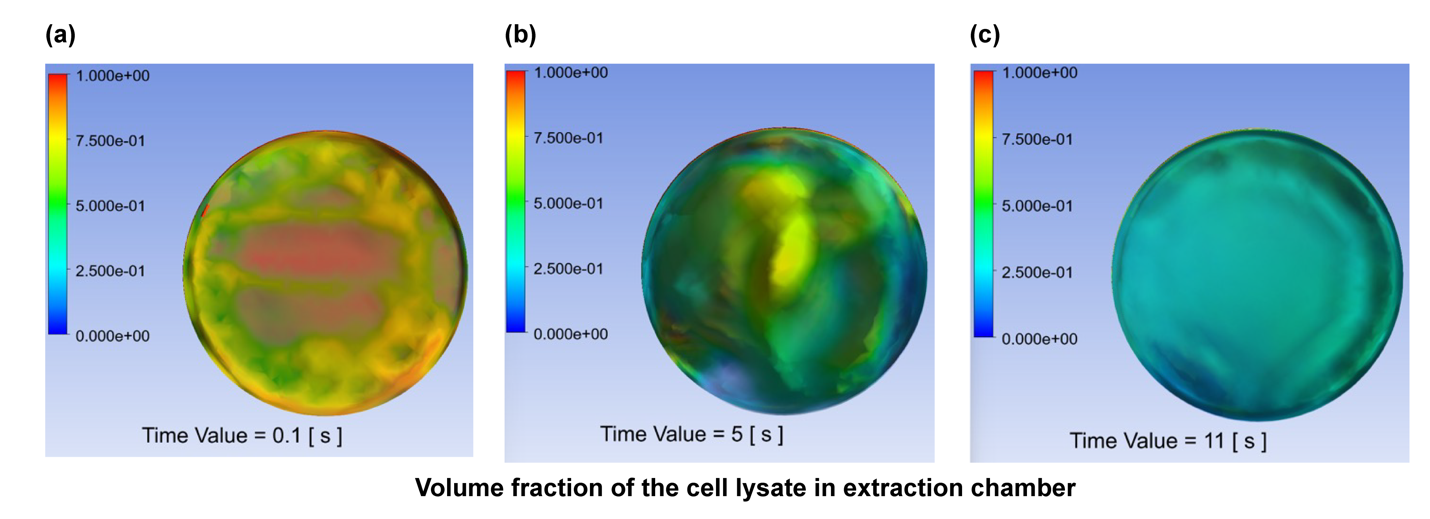


Figure S8 (a) Adding lysis buffer into lysis chambers: Solenoid valves S1 and S2 are opened. Rotary valves R1 and R2 are opened. External positive pressure is applied to the lysis buffer tube through S1, driving the buffer into 24 lysis chambers via S2 and R1. S1 is then closed, and gas is injected into S2 to completely push the residual buffer in the pipeline into the lysis chambers. R2 is opened to facilitate timely evacuation of gas in the lysis chambers, preventing buffer entry failure due to excessive internal pressure. (b) Transferring lysis products to extraction chambers: S1 and R2 are closed, and the α valve and R5 are opened. Gas is injected into the lysis chambers through S2 to push the lysis products into the extraction chambers via channel I. Solid lines indicate unobstructed flow paths, dashed lines indicate closed flow paths, and highlighted parts indicate ongoing steps.


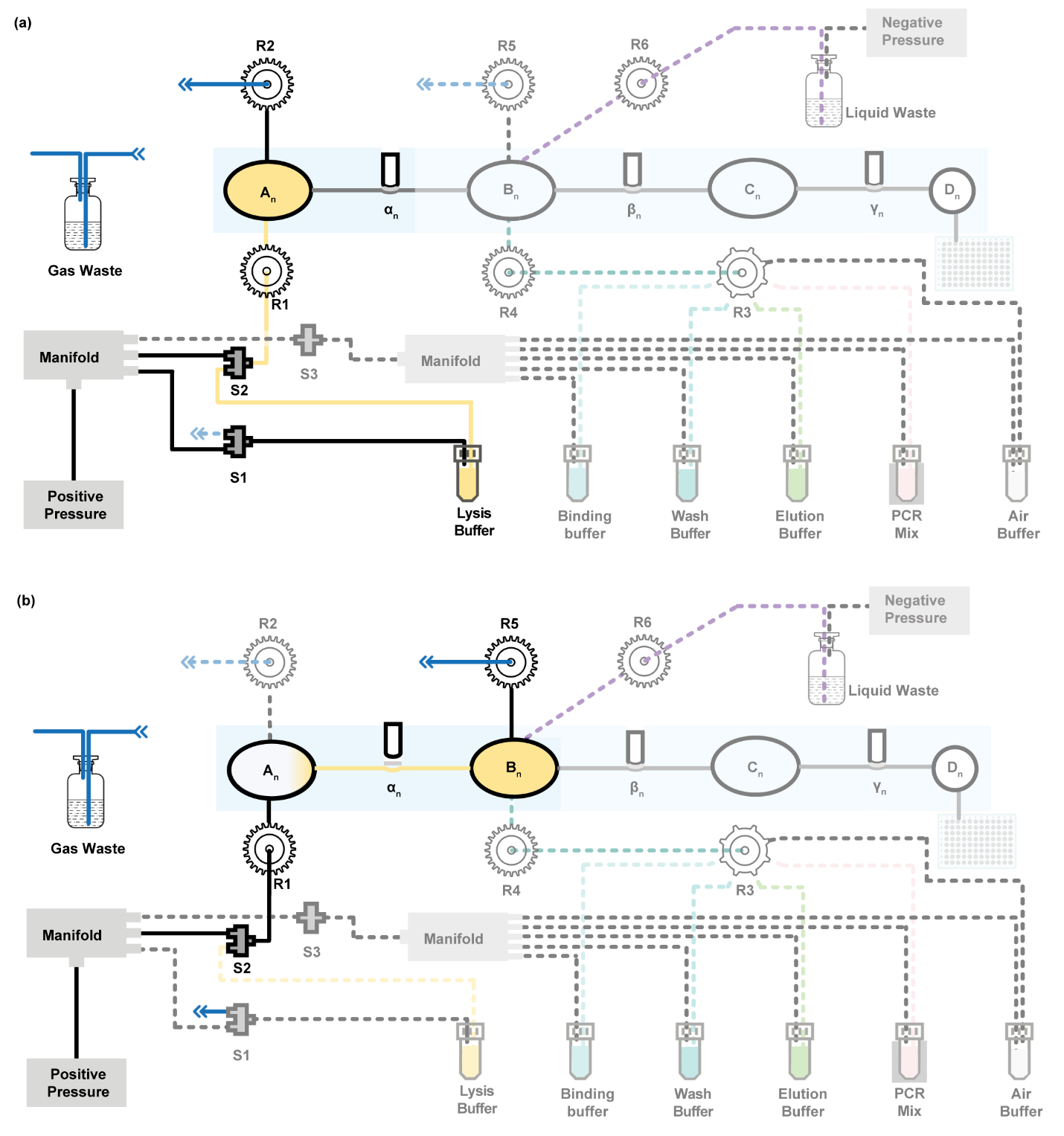


**
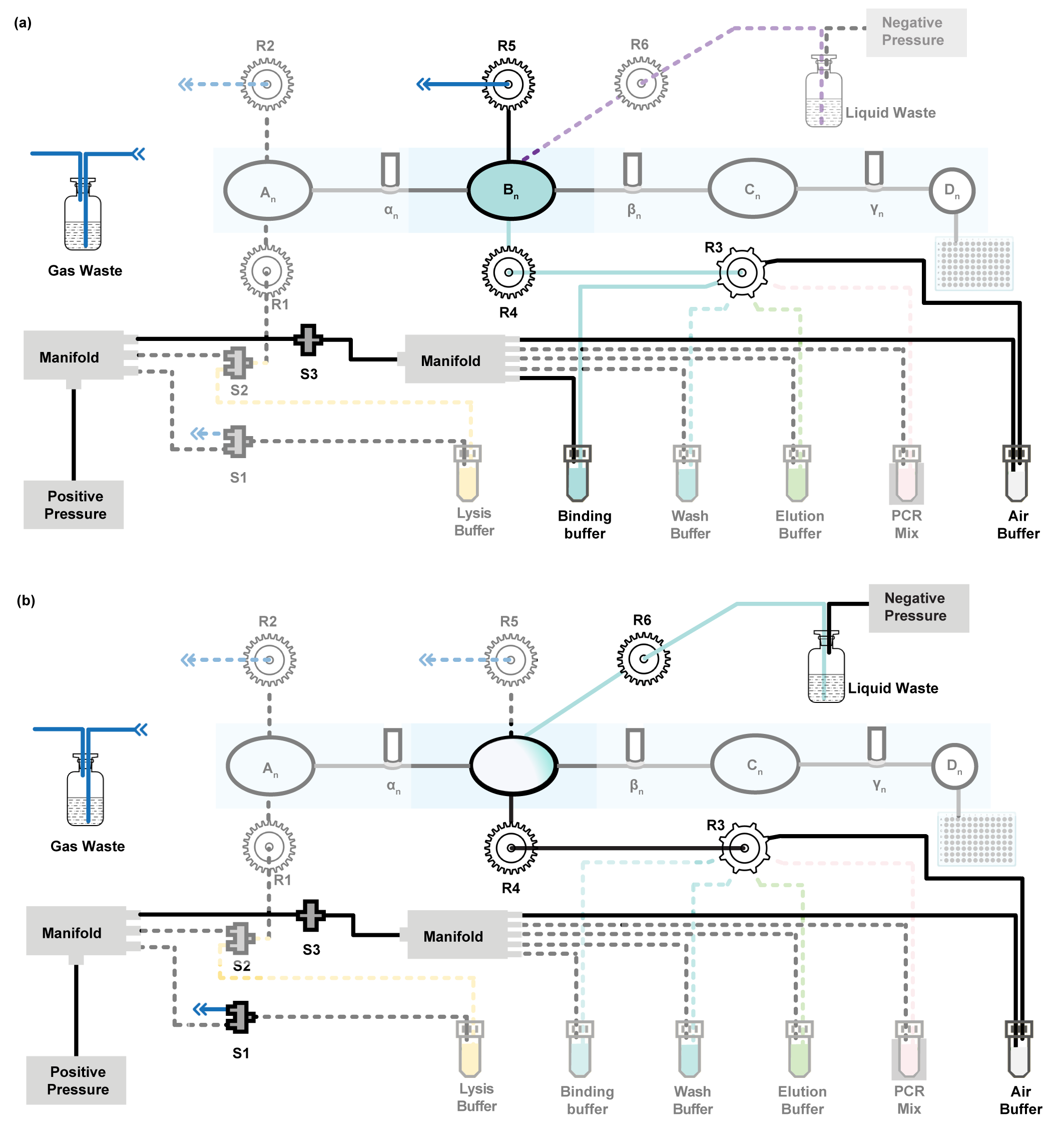
**Figure S9 Extraction operation. (a), inject binding buffer. Close α and β valves, open solenoid S3 and rotate R3 to binding buffer port (R3-P1). Rotate R3 to air port to inject air to push buffer completely into extraction chamber. During the injection, keep the exhausting line clear (R5) and close waste pipeline (R6). (b) Discharge waste effluent. Keep α and β valves closed, rotate R3 to air port (R3-P5) and close R5, open R6 to push the waste liqiud out. Dash line means the pipelines are blocked. Repeat the above steps for different extration buffer by changing the injection port of R3(1-3).


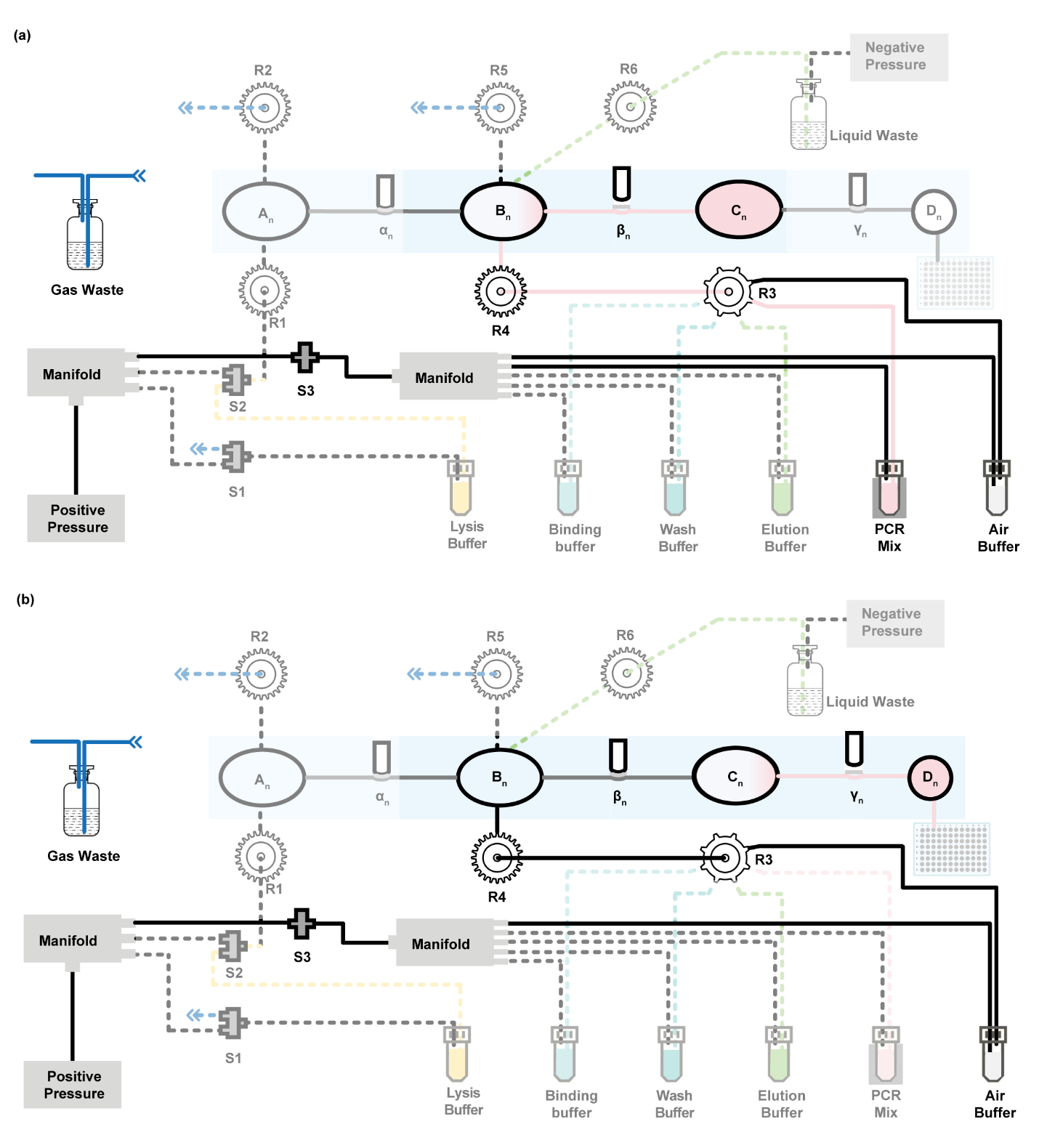


Figure S10 (a) Inject PCR mix. Rotate R3 to PCR mix port (P4), close R5 and R6, open β valve to inject PCR mix. Then rotate R3 to air port (P5) to push PCR mix completely into amplification chamber. During amplification time, close β and γ valves to reduce evaporation. (b) Export PCR production. Rotate R3 to air port (P5), close R5 and R6, open β and γ valves to push the production out. Dash line means the pipelines are blocked.

Figure S11 Consistency test of DNA extraction in 24-sample microfluidic system. (a) **Nucleic acid extraction yield across 24 individual channels**: The bar chart shows the nucleic acid extraction yield (ng) for each of the 24 channels. The yields were 2804.59 ng ± 87.82 ng (mean ± SD, n = 3), with the coefficient of variation (CV) of approximately 3.14%, demonstrating the excellent reproducibility and uniformity of the extraction process across all channels. Error bars represent the standard deviation of triplicate measurements for each channel. (b) **Agarose gel electrophoresis of TFRC amplicons**: All 24 channels produced clear, distinct bands of the expected size (~200 bp) following amplification of the TFRC gene, confirming successful and consistent nucleic acid amplification from the extracted products. The uniform band intensity further validates the high consistency of the 24-channel chip performance.


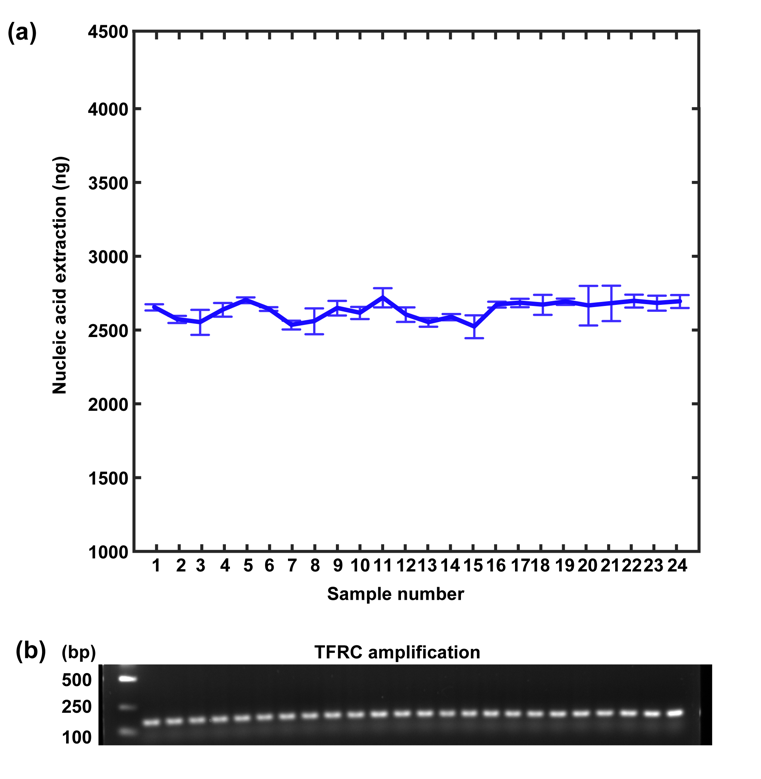

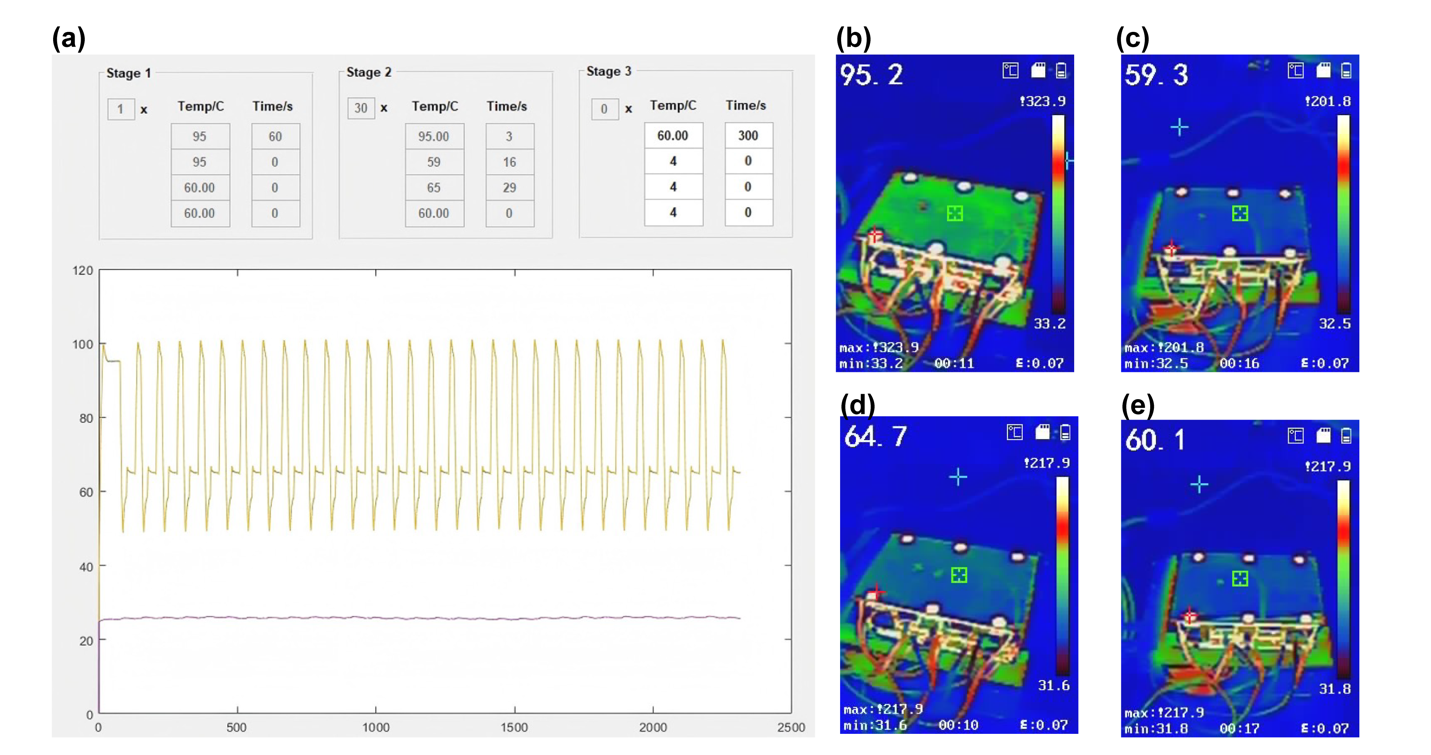


Figure S12 **Temperature uniformity and performance of the 24-channel thermal control module.** (a) **Thermal cycling profile and program:** The top panel shows the three-stage temperature program (Stage 1: 95 °C for 60 s; Stage 2: 95 °C for 3 s, 59 °C for 16 s, 65 °C for 29 s; Stage 3: 60 °C for 300 s). The bottom panel displays the real-time temperature trace (gold line) during PCR thermal cycling, demonstrating stable and reproducible temperature transitions, with a reference trace (purple line) for comparison. (b–e) **Infrared thermal imaging of the thermal control module:** Representative thermal images captured at set temperatures of (b) 95 °C, (c) 59 °C, (d) 65 °C, and (e) 60 °C. The color scale represents temperature distribution, and the uniform coloration across the entire module surface confirms excellent temperature homogeneity between all 24 channels, with minimal temperature variation (± 0.1 °C) regardless of the setpoint.

**
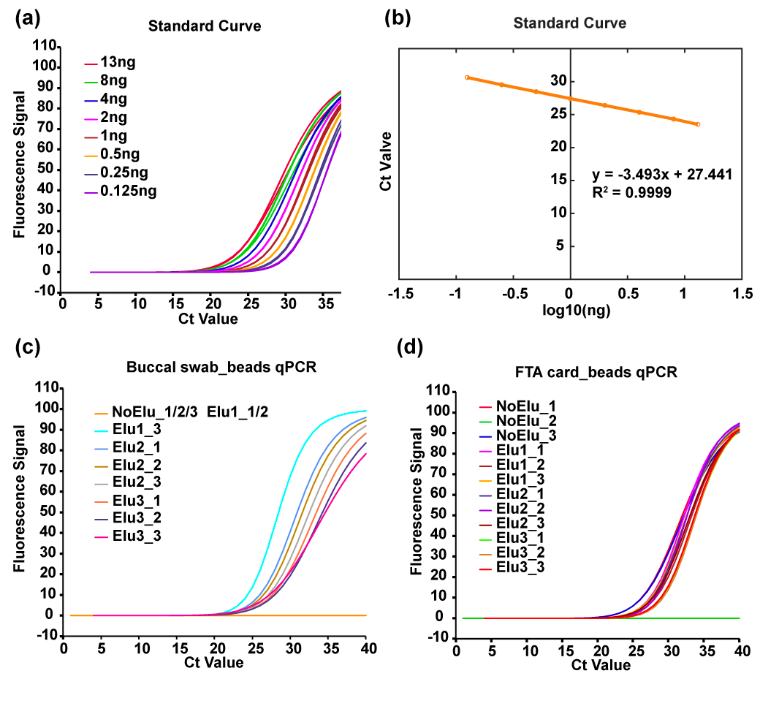
**

Figure S13 Elution times test for optimal concentration of DNA for STR amplification. (a-b) Standard curve of DNA input and Ct value. (c) Elution times test for buccal swab samples. (d) Elution times test for FTA card samples.

Figure S14 The STR profiles of the buccal swab sample and the FTA card sample from two donors were consistent after automated nucleic acid extraction and amplification by the system.


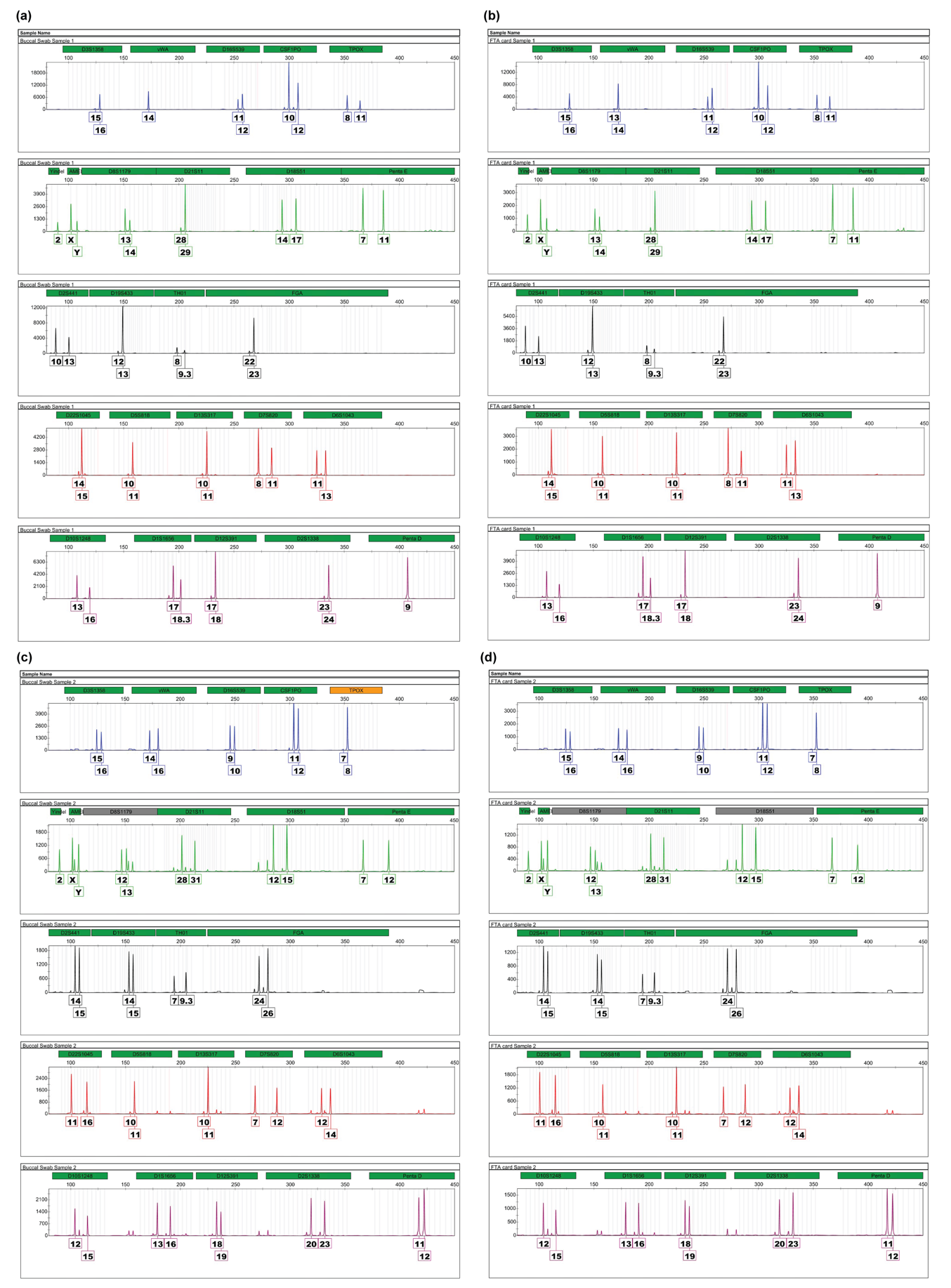

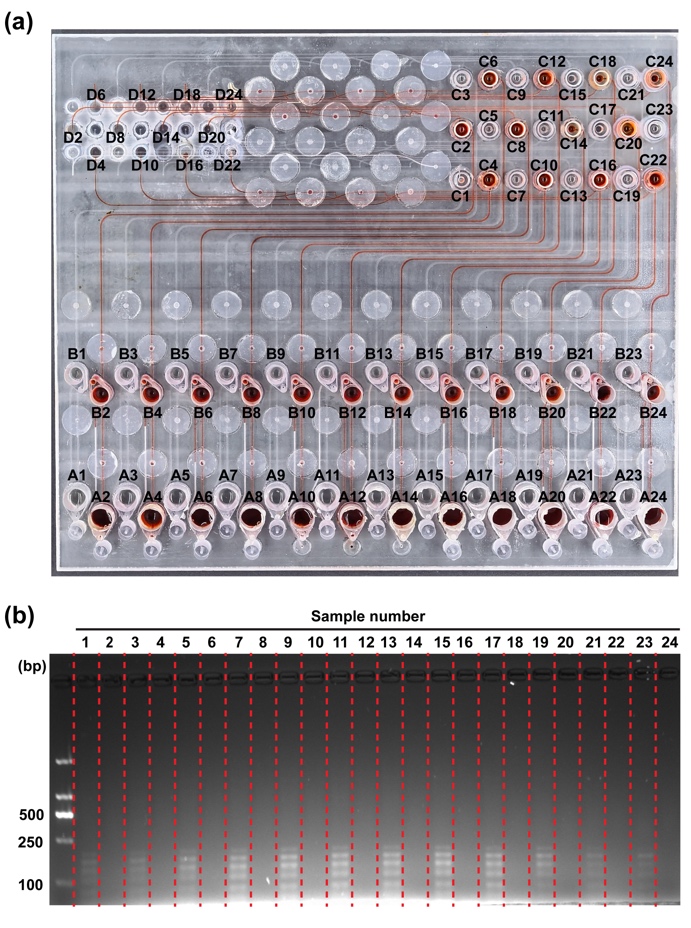


Figure S15 **Demonstration of channel independence and absence of cross-contamination in the 24-channel microfluidic chip.** (a) **Isolation of adjacent channels**: interval filling of colored reagents and transparent water into alternating channels showed no leakage or mixing, confirming physical isolation. (b) **PCR validation**: Agarose gel electrophoresis of amplicons showed target bands only in sample-loaded channels, with no bands in adjacent blank controls, verifying no cross-contamination during processing and amplification.


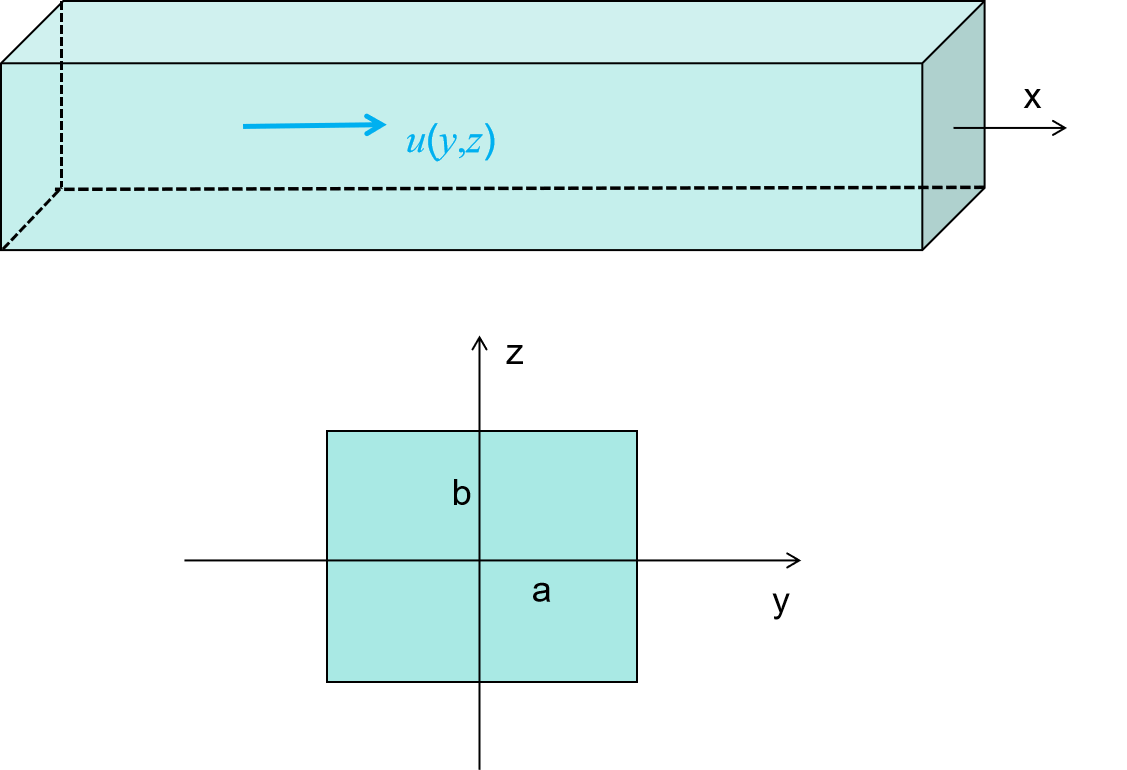


Figure S16 Schematic diagram of a single flow channel on the chip. u(y,z) represents the fluid direction. b represents half height of the cross section of the channel, a represents half length of the cross section of the channel.

### **Tables**

**
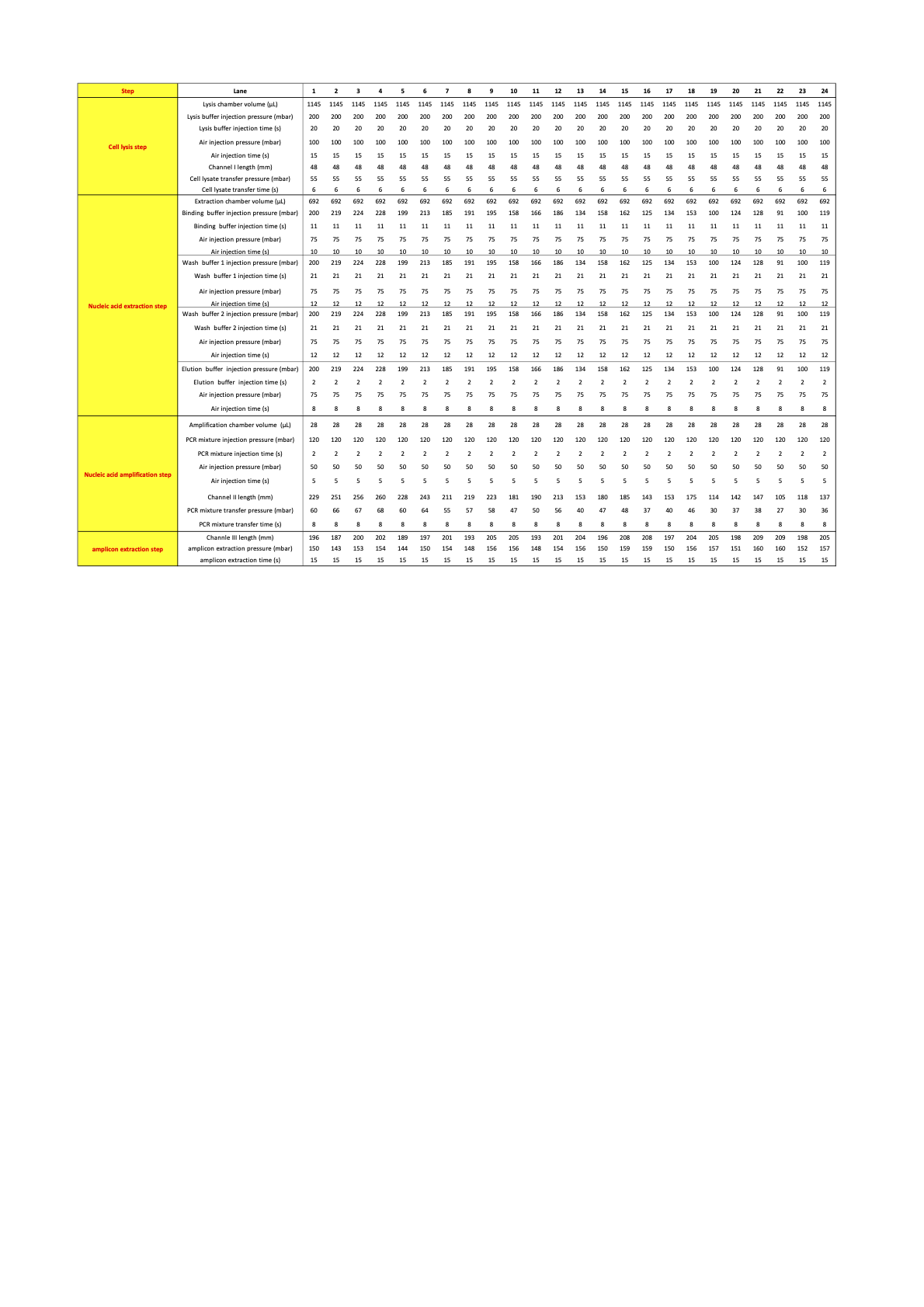
Table S1 Length of the Channels and the applied parameters for 24-channel chip**

**Table S2 24-sample DNA extractions**

| Sample number | Average of DNA extraction (ng) | Mean Standard deviation (ng) | Average coefficient of variation (%) |
| --- | --- | --- | --- |
| 1 | 2837.67 | 38.55 | 1.36 |
| 2 | 2693.78 | 43.60 | 1.62 |
| 3 | 2659.11 | 149.10 | 5.61 |
| 4 | 2809.11 | 83.00 | 2.95 |
| 5 | 2922.00 | 35.59 | 1.22 |
| 6 | 2817.33 | 22.50 | 0.80 |
| 7 | 2627.11 | 53.15 | 2.02 |
| 8 | 2672.00 | 155.20 | 5.81 |
| 9 | 2828.89 | 87.58 | 3.10 |
| 10 | 2769.89 | 72.05 | 2.60 |
| 11 | 2951.33 | 114.50 | 3.88 |
| 12 | 2750.33 | 87.75 | 3.19 |
| 13 | 2658.78 | 53.43 | 2.01 |
| 14 | 2722.22 | 37.50 | 1.38 |
| 15 | 2606.00 | 137.39 | 5.27 |
| 16 | 2869.44 | 36.30 | 1.27 |
| 17 | 2890.00 | 49.24 | 1.70 |
| 18 | 2868.33 | 118.99 | 4.15 |
| 19 | 2906.11 | 39.10 | 1.35 |
| 20 | 2858.00 | 235.22 | 8.23 |
| 21 | 2886.00 | 212.94 | 7.38 |
| 22 | 2912.00 | 77.70 | 2.67 |
| 23 | 2886.67 | 90.05 | 3.12 |
| 24 | 2908.00 | 77.12 | 2.65 |
| average | 2804.59 | 87.82 | 3.14 |

###

| DNA input (ng) | Amplification success rate (%) | Average peak height (RFU) | Average peak-height balance (P, %) | Standard deviation (%) |
| --- | --- | --- | --- | --- |
| 0.125 | 100 | 2,150 ± 320 | 62 ± 4 | 4.1 |
| 0.5 | 100 | 3,890 ± 410 | 71 ± 3 | 3.2 |
| 1 | 100 | 5,230 ± 380 | 75 ± 2 | 2.5 |
| 2 | 100 | 6,780 ± 450 | 78 ± 2 | 2.1 |
| 4 | 100 | 8,120 ± 520 | 80 ± 1 | 1.7 |
| 6 | 100 | 8,950 ± 490 | 82 ± 1 | 1.4 |
| 8 | 100 | 9,340 ± 510 | 83 ± 1 | 1.2 |
| 10 | 100 | 9,760 ± 480 | 84 ± 1 | 1.1 |

**Table S3 Temperature determination for temperature control module**

| Temperature setpoint (°C) | Actual temperature (°C, Mean, n=3) | Standard deviation (SD, °C) |
| --- | --- | --- |
| 95 (Denaturation) | 94.9 | 0.3 |
| 59 (Annealing) | 59.1 | 0.2 |
| 65 (Extension) | 64.9 | 0.2 |
| 60 (Final Extension) | 60.1 | 0.1 |

**Table S4 Average peak-height balance (P) across STR loci by DNA input concentration**

**Table S5 Comparison of two commercial products and our system^1^.**

| comparison | | 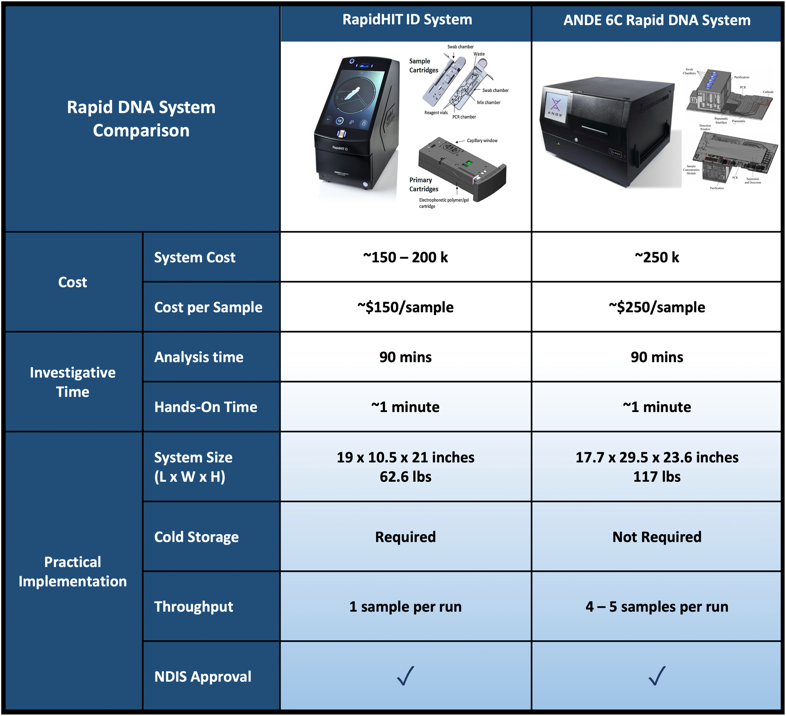RapidHIT ID system | ANDE 6C Rapid DNA system  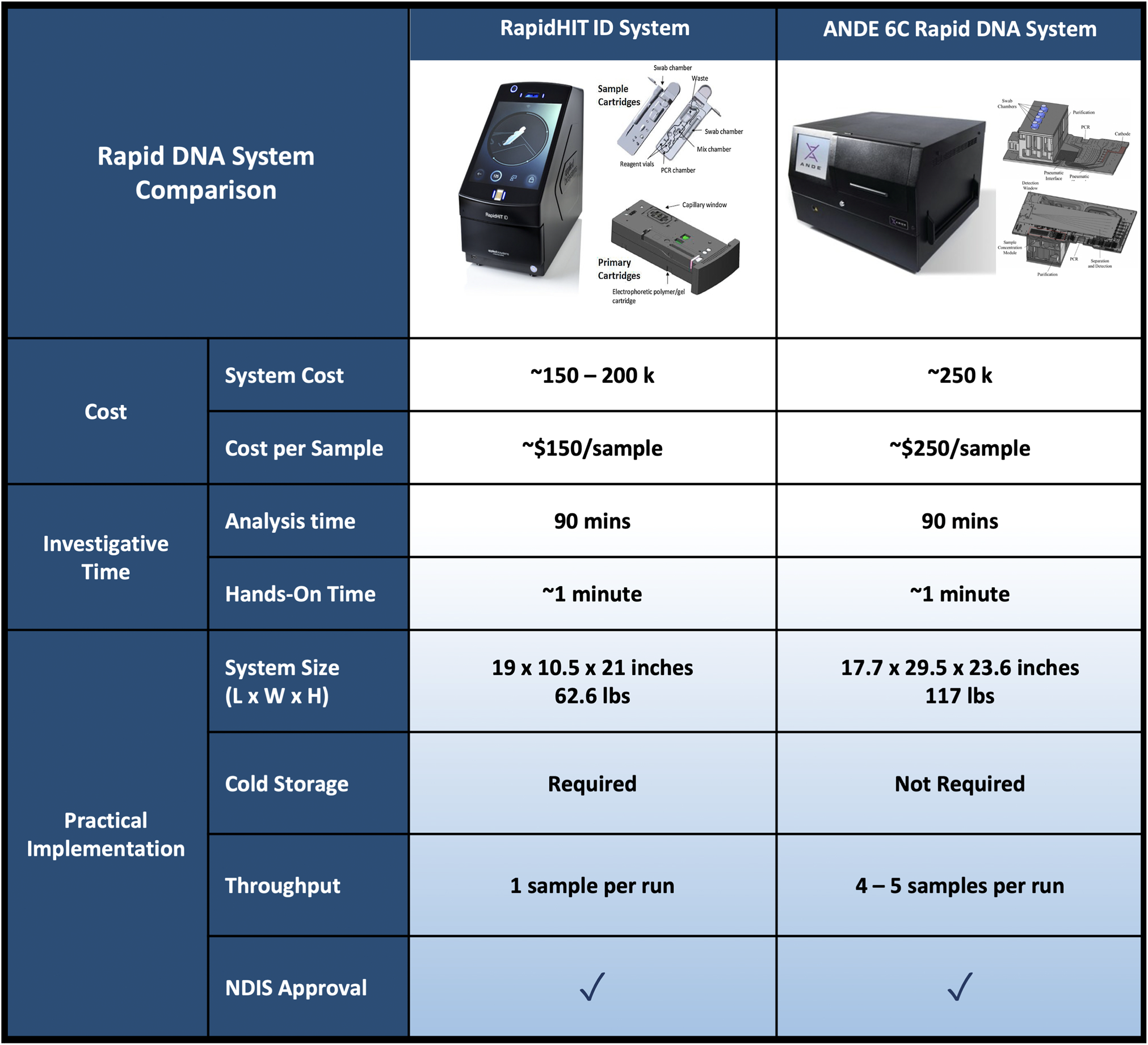 | our system  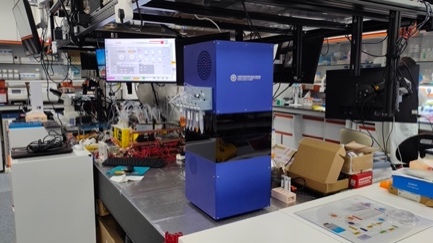 |
| --- | --- | --- | --- | --- |
| cost | System cost | ~$153 k | ~$142.5 k | ~$100 k |
|  | Cost per sample | ~$212 /sample | ~$285 /sample | ~$50/sample, Custom PC chip: ~$15, Reagents: ~$20, FTA card / buccal swab: ~$1 |
| Investigative time | Analysis time | 90 mins/sample | 90 mins/sample | 30 mins/sample |
|  | Hands-on time | ~1 min | ~1 min | ~1 min |
| Practical implementation | throughput | 1 sample per run | 4-5 samples per run | 24 samples per run |
|  | Sample type | Buccal | Buccal &FTA | Buccal &FTA |
|  | Minimal input | 0.62 ng | 0.155 ng | 0.125 ng |
|  | Cell lysis | N/A | N/A | Detergent and heat |
|  | DNA purification | N/A | N/A | Magnetic beads |
|  | DNA amplification | PCR | PCR | PCR |
|  | Sample exportable | no | no | yes |
| appearance | Chip size  (L x W x H) | N/A | 29.5 x 16.5 x 9.3 cm | 25 x 21.5 x 0.5 cm |
|  | System size (L x W x H) | 48 x 53 x 27 cm | 45 x 75 x 59 cm | 30 x 30 x 78 cm |
|  | weight | 28.4 kg | 54.4 kg | 25 kg |
|  | portability | yes | no | yes |
| consumables | | Primary Cartridge (≥100 runs)  RapidHIT™ ID ACE Sample Cartridge  (reference sample)  RapidINTEL™ Sample Cartridge  (crime scene samples) | ANDE® swab  A-Chip (reference samples)  I-Chip (crime scene samples) | 24-sample Chip |
| Stage | | commercial | commercial | prototype |
| Software | | RapidLINK^TM^ Software | ANDE Expert System  ANDE FAIRS^TM^ Software | Self-developed |

**Table S6 qPCR results for standard samples**

| Ct Value | Input (ng) | log10(input) |
| --- | --- | --- |
| 23.514 | 13 | 1.113943 |
| 24.32 | 8 | 0.90309 |
| 25.364 | 4 | 0.60206 |
| 26.391 | 2 | 0.30103 |
| 27.427 | 1 | 0 |
| 28.474 | 0.5 | -0.30103 |
| 29.52 | 0.25 | -0.60206 |
| 30.625 | 0.125 | -0.90309 |

**Table S7 qPCR results for eluted beads from buccal swab samples**

| Elution time | Ct Value | Input (ng) | SD |
| --- | --- | --- | --- |
| No Elution | Undetermined | Undetermined | Undetermined |
|  | Undetermined | Undetermined |  |
|  | Undetermined | Undetermined |  |
| Elute once | Undetermined | Undetermined | Undetermined |
|  | Undetermined | Undetermined |  |
|  | 24.235 | 8.276289 |  |
| Elute twice | 25.8 | 2.949828 | 0.78203299 |
|  | 26.301 | 2.120152 |  |
|  | 26.945 | 1.38675 |  |
| Elute three times | 27.475 | 0.977837 | 0.25142895 |
|  | 27.757 | 0.811958 |  |
|  | 27.036 | 1.306009 |  |

**Table S8 qPCR results for eluted beads from FTA card samples**

| Elution time | Ct Value | Input (ng) | SD |
| --- | --- | --- | --- |
| No Elution | 26.036 | 2.126024 | 0.03761101 |
|  | Undetermined | Undetermined |  |
|  | 26.075 | 2.072834 |  |
| Elute once | 27.241 | 0.97182 | 0.21088751 |
|  | 28.025 | 0.583963 |  |
|  | 27.323 | 0.921404 |  |
| Elute twice | 27.553 | 0.793518 | 0.0942555 |
|  | 27.955 | 0.611133 |  |
|  | 27.653 | 0.743606 |  |
| Elute three times | 28.973 | 0.315438 | 0.03273684 |
|  | 29.083 | 0.293682 |  |
|  | 28.778 | 0.35804 |  |

1 Dalin, E., Seidlitz, H., Ansell, R. & Forsberg, C. Rapid DNA: A summary of available Rapid DNA systems. 25 (National Forensic Centre, Sweden, 2022).
